# ﻿Eleven species of jumping spiders from Sichuan, Xizang, and Yunnan, China (Araneae, Salticidae)

**DOI:** 10.3897/zookeys.1192.114589

**Published:** 2024-02-21

**Authors:** Cheng Wang, Xiaoqi Mi, Shuqiang Li

**Affiliations:** 1 Guizhou Provincial Key Laboratory for Biodiversity Conservation and Utilization in the Fanjing Mountain Region, Tongren University, Tongren, Guizhou 554300, China Tongren University Tongren China; 2 Institute of Zoology, Chinese Academy of Sciences, Beijing 100101, China Institute of Zoology, Chinese Academy of Sciences Beijing China

**Keywords:** New combination, salticid, southwestern China, taxonomy

## Abstract

Ten new species of jumping spiders are described from China, including *Attulusjimani***sp. nov.** (♂♀) from Yunnan, *Colaxescibagou***sp. nov.** (♂♀), *Epeuspengi***sp. nov.** (♂♀), *Evarchazayu***sp. nov.** (♂♀), *Iciuszang***sp. nov.** (♂♀), *Pancoriusnyingchi***sp. nov.** (♂♀), *Stertiniusliqingae***sp. nov.** (♂♀), and *Synagelidesmedog***sp. nov.** (♀) from Xizang, *S.tianquan***sp. nov.** (♂♀), and *Yaginumaellaerlang***sp. nov.** (♂♀) from Sichuan. The hitherto unknown female of *Phintellalongapophysis* Lei & Peng, 2013 is described for the first time. Diagnostic photos and the distributional maps for all species are provided. Four new combinations are proposed: *Epeusdilucidus* (Próchniewicz, 1990), **comb. nov.**, and *E.guangxi* (Peng & Li, 2002), **comb. nov.** transferred from *Plexippoides* Prószyński, 1984, *Phintellasufflava* (Jastrzębski, 2009), **comb. nov.** transferred from *Carrhotus* Thorell, 1891, and *Yaginumaellaarmata* (Jastrzębski, 2011), **comb. nov.** transferred from *Pancorius* Simon, 1902.

## ﻿Introduction

With the series of taxonomic studies conducted, the knowledge of the family Salticidae Blackwall, 1841 from China has significantly increased, and the recorded species number has exceeded 720, which is higher than Brazil, the most species-richest country worldwide (Metzner 2024; WSC 2024). However, the Chinese jumping spider remains a poor species survey, and in the light of prospection by Li (2020), the species number could reach ca 1500.

Sichuan, Xizang, and Yunnan are the three bordered provinces in southwestern China. They partly belong to the Hengduan Mountains and Himalayan Mountains, which have been the centre of diversification for several spider groups and have presented a very high species diversity of jumping spiders, especially Yunnan, where at least 235 species are documented, far exceeding the number of salticid species known from Vietnam (161), Japan (150), and about 2/3 the species number known from India (364), and 3/5 known from Indonesia (397) (Wang and Li 2021; Li and Lin 2024; Metzner 2024; WSC 2024).

In our recent examination of jumping spider specimens collected from those three provinces, ten species were recognized as new to science, and the unknown females of *Phintellalongapophysis* Lei & Peng, 2013 were also found. This work aims to describe the new species, the unknown female of *P.longapophysis* and propose four new combinations in other salticids.

## ﻿Material and methods

Specimens were collected by beating shrubs or hand collecting. They were preserved in 80% or absolute ethanol. Specimens are deposited in the Institute of Zoology, Chinese Academy of Sciences in Beijing (IZCAS), China, and Tongren University (TRU) in Tongren, China. The specimens were examined with an Olympus SZX10 stereomicroscope. After dissection, the vulva was cleared in trypsin enzyme solution before examination and imaging. Images of the copulatory organs and habitus were taken with a Kuy Nice CCD mounted on an Olympus BX43 compound microscope. Compound focus images were generated using Helicon Focus v. 6.7.1. Drawings of the paths of copulatory ducts were generated by Adobe Illustrator CC 2018. All measurements are given in millimetres. Leg measurements are given as total length (femur, patella, tibia, metatarsus, tarsus). Abbreviations used in the text and figures are as follows:

**ALE** anterior lateral eye; **AME** anterior median eye; **AERW** anterior eye row width; **AR** atrial ridge; **AS** anterior chamber of spermatheca; **At** atrium; **CD** copulatory duct; **CO** copulatory opening; **E** embolus; **EFL** eye field length; **FD** fertilization duct; **H** epigynal hood; **LP** lamellar process; **MA** median apophysis; **MS** median septum; **PCA** prolateral cymbial apophysis; **PERW** posterior eye row width; **PL** posterior lobe; **PLE** posterior lateral eye; **PS** posterior chamber of spermatheca; **RCA** retrolateral cymbial apophysis; **RTA** retrolateral tibial apophysis; **S** spermatheca; **SD** sperm duct; **TF** tegular flap.

Institutional abbreviations: **IZCAS** Institute of Zoology, Chinese Academy of Sciences; **TRU** Tongren University.

## ﻿Taxonomy

**Family Salticidae Blackwall, 1841**

### ﻿Genus Attulus

Taxon classificationAnimaliaAraneaeSalticidae

Simon, 1889

A3CACEBB-54FB-5A56-8FBE-4F5DE2908F60

#### Type species.

*Attushelveolus* Simon, 1871.

#### Comments.

*Attulus* is placed in the Subtribe Sitticina Simon, 1901, together with five other genera (Maddison et al. 2020; Metzner 2024) and represented by 59 nominal species widely distributed in Eurasia (WSC 2024). It can be easily distinguished from other genera of the Subtribe except *Sittisax* Prószyński, 2017 based on the long fourth legs and absence of retromarginal cheliceral teeth (Maddison et al. 2020), and it can be distinguished from *Sittisax* by the tube-shaped, folded spermathecae.

### ﻿ Attulus jimani sp. nov.

Taxon classificationAnimaliaAraneaeSalticidae

228A77E8-CB70-54A3-B53B-A3D10FC222C5

https://zoobank.org/0DA85789-371E-4548-A0C6-C7391BF8FD63

Figs 1 , 2 , 22A

#### Type material.

***Holotype*** ♂ (IZCAS-Ar44763), China: Yunnan: Deqen County (28°27.88′N, 98°54.98′E, ca 3350 m), 5 Jun. 1994, J. He leg. ***Paratypes*** 4♂2♀ (IZCAS-Ar44764–44769), same data as for holotype.

#### Etymology.

The specific name is after the collector, Jiman He; noun (name) in genitive case.

#### Diagnosis.

**Figure 1.**
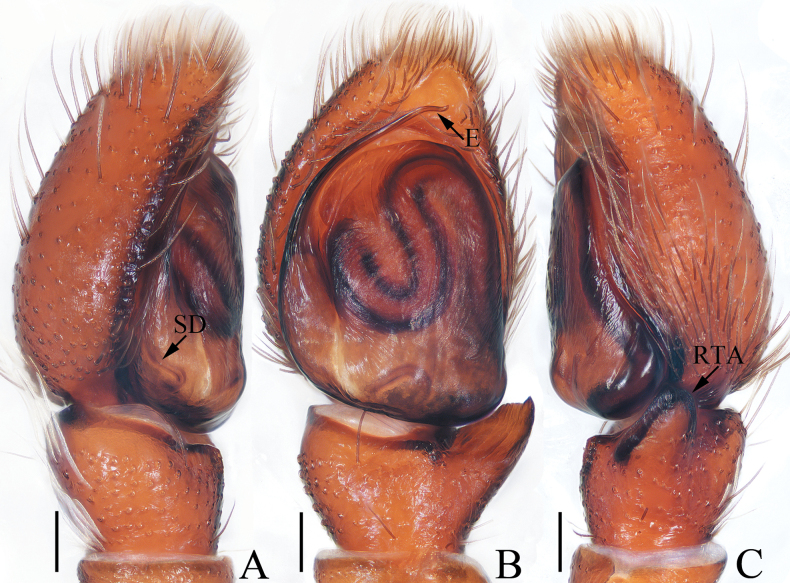
Male palp of *Attulusjimani* sp. nov., holotype **A** prolateral **B** ventral **C** retrolateral. Scale bars: 0.1 mm.

**Figure 2.**
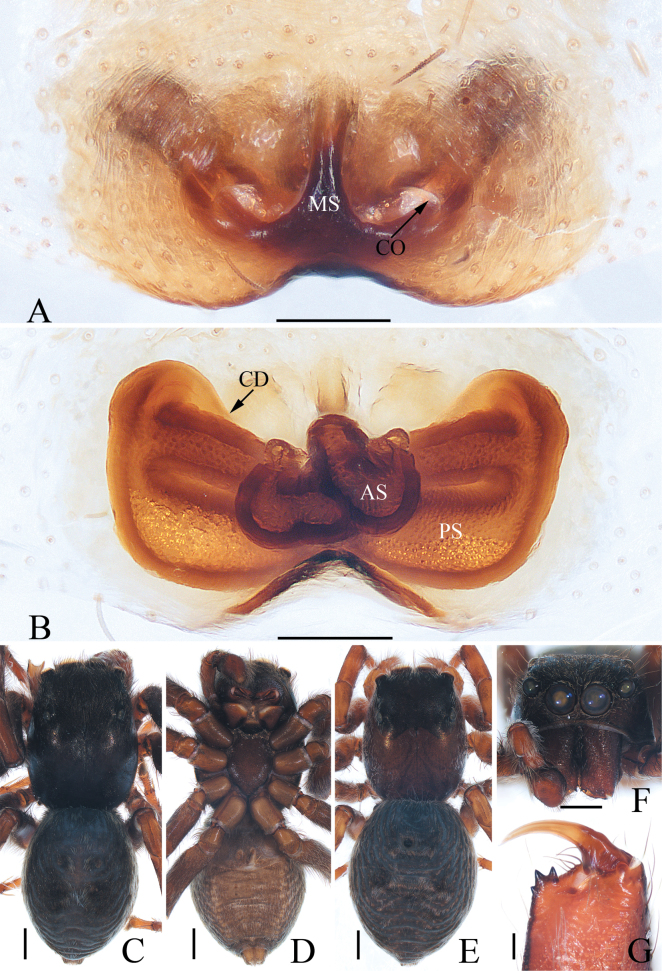
*Attulusjimani* sp. nov., male holotype and female paratype **A** epigyne, ventral **B** vulva, dorsal **C** holotype habitus, dorsal **D** ditto, ventral **E** female paratype habitus, dorsal **F** holotype carapace, frontal **G** holotype chelicera, posterior. Scale bars: 0.1 mm (**A, B, G**); 0.5 mm (**C–F**).

The male of *Attulusjimani* sp. nov. resembles that of *A.dubatolovi* (Logunov & Rakov, 1998) in the general shape of palp, especially the RTA, but it differs as follows: 1) embolus originating at ca 8:30 o’clock position (Fig. 1A, B), versus about 6 o’clock position in *A.dubatolovi* (Logunov and Rakov 1998: fig. 74); 2) RTA blunt apically in retrolateral view (Fig. 1C), versus sharply pointed in *A.dubatolovi* (Logunov and Rakov 1998: fig. 75). The female of *A.jimani* sp. nov. closely resembles that of *A.clavator* (Schenkel, 1936) in the general shape of epigyne and vulva, but it can be distinguished by the spermatheca having an elongated anterior chamber, and a transversely extending posterior chamber, and by the absence of markings on the dorsum of abdomen (Fig. 2B, E), versus the spermatheca having a spherical anterior chamber, and posterolaterlly extending posterior chamber, and the presence of a pair of oval spots on the dorsum of abdomen in *A.clavator* (Peng 2020: fig. 302a, e). The female also somewhat resembles that of *A.nitidus* (Hu, 2001) but is readily distinguished by the median septum, which is separated from epigastric furrow about one-third its length and almost equal in width anteromedially (Fig. 2A), versus at least half its length, and widened anteriorly in *A.nitidus* (Hu 2001: fig. 266-3).

#### Description.

**Male** (Figs 1, 2C, D, F, G). Total length 4.81. Carapace 2.45 long, 1.86 wide. Abdomen 2.50 long, 1.93 wide. Eye sizes and inter-distances: AME 0.37, ALE 0.24, PLE 0.20, AERW 1.40, PERW 1.33, EFL 0.90. ***Legs***: I 6.21 (1.75, 1.13, 1.60, 1.15, 0.58), II 4.86 (1.45, 0.88, 1.18, 0.85, 0.50), III 3.91 (1.15, 0.60, 0.93, 0.78, 0.45), IV 5.49 (1.75, 0.78, 1.25, 1.08, 0.63). Carapace dark brown, covered with dense setae on the elevated cephalon; fovea dark, longitudinal. Chelicerae red-brown to dark brown, each with three teeth on promargin. Endites widened at distal half, with pale antero-inner areas. Labium dark, tapered, with dark grey anterior margin. Sternum red-brown to dark brown, about 1.5 times longer than wide. Legs red-brown to dark brown. Abdomen oval, dorsum dark brown, without distinct markings, with three pairs of muscle depressions medially, covered with pale, thin setae; venter grey-brown, covered with dense thin setae.

***Palp*** (Fig. 1A–C): tibia slightly wider than long, with flat and broad RTA almost shovel-shaped in ventral view; cymbium less than 1.5 times longer than wide, setose; bulb flat, almost oval; embolus originating at ca 8:30 o’clock position of bulb, widened at base, and followed by the slender remaining portion slightly curved and ending with blunt tip.

**Female** (Fig. 2A, B, E). Total length 5.06. Carapace 2.35 long, 1.82 wide. Abdomen 2.78 long, 2.23 wide. Eye sizes and inter-distances: AME 0.38, ALE 0.25, PLE 0.20, AERW 1.39, PERW 1.39, EFL 0.86. ***Legs***: I 4.18 (1.25, 0.83, 1.00, 0.65, 0.45), II 3.81 (1.13, 0.75, 0.88, 0.60, 0.45), III 3.72 (1.13, 0.63, 0.83, 0.68, 0.45), IV 5.46 (1.80, 0.70, 1.38, 1.08, 0.50). ***Habitus*** (Fig. 2E) similar to that of the male except paler in colour.

***Epigyne and vulva*** (Fig. 2A, B): wider than long, atrium irregular, posteromedially located, separated by the arch-bridge-shaped median septum; copulatory openings almost half-round, situated at the lateral sides of the base of median septum, far away from each other about 1/3 the epigynal width; copulatory ducts anterolaterally extending before strongly curved about 150° at distal end; spermathecae divided into two elongated chambers, the posterior chamber transversely extending.

#### Distribution.

Known only from the type locality in Yunnan, China (Fig. 22A).

### ﻿Genus Colaxes

Taxon classificationAnimaliaAraneaeSalticidae

Simon, 1900

C988BCD6-AFB0-5F94-98B2-2560CC2091EA

#### Type species.

*Colaxesnitidiventris* Simon, 1900.

#### Comments.

*Colaxes* is a rather poorly known genus, which is placed in the tribe Ballini Banks, 1892 together with 21 other genera, and only contains four endemic species recorded from India and Sri Lanka (Maddison 2015; Metzner 2024; WSC 2024). The genus was diagnosed by Benjamin (2004) for the following: 1) the presence of dark markings on the laterals of the abdomen and the absence of markings on the lateral sides of legs I–IV; 2) the presence of only four spines on tibia I (except for *Ballus* C. L. Koch, 1850); and 3) absence of leaf-like setae ventrally on tibiae I (except for *Cynapes* Simon, 1900 and *Ballus*). However, the mentioned diagnosis was doubted by Paul et al. (2020), who also pointed out that the taxonomic validity of *Colaxes* requires further investigation. It is worth noting that Paul et al. (2020) were not concerned about the absence of leaf-like setae ventrally on tibiae I, an essential character in Benjamin’s taxonomic study of the tribe Ballini in 2004. According to this character, *Colaxes* can be easily distinguished from Asian Ballini genera except *Ballus*, *Copocrossa* Simon, 1901, and *Mantisatta* Warburton, 1900.

Moreover, *Colaxes* can be distinguished from *Ballus* by the carapace, which is longer than wide, but wider than long in the latter (Benjamin 2004), and it can be distinguished from *Copocrossa*, and *Mantisatta* by lacking much-developed leg I (see the colour habitus photos of *Copocrossatenuilineata* (Simon, 1900) and *Mantisattatrucidans* Warburton, 1900 in Metzner 2024). However, a proper definition of the genus can’t be provided because the generotype is relatively poorly known, and members are rather diverse in habitus and copulatory organs. The below new species might not be true *Colaxes*. However, we still decided to temporarily assign it to the genus because it lacks leaf-like setae ventrally on tibiae I, shares similar copulatory organs with the known member, *C.horton* Benjamin, 2004, and is geographically adjacent to the generotype.

### ﻿ Colaxes cibagou sp. nov.

Taxon classificationAnimaliaAraneaeSalticidae

AAD9E17E-8D58-531D-90E8-332C53C80056

https://zoobank.org/3E3387C2-5AA8-4A75-B079-EDA98189747C

Figs 3 , 4 , 22B

#### Type material.

***Holotype*** ♂ (TRU-XZ-JS-0001), China: Xizang: Zayu County, Cibagou National Nature Reserve (28°41.43′N, 97°2.86′E, ca 2570 m), 26 Jun. 2023, C. Wang leg. ***Paratypes*** 2♂1♀ (TRU-XZ-JS-0002–0004), same data as for holotype; 3♂2♀ (TRU-XZ-JS-0005–0009), same locality as for holotype, 13 Aug. 2003, C. Wang & H. Yao leg.

#### Etymology.

The species name is a noun derived from the type locality: Cibagou National Nature Reserve.

#### Diagnosis.

**Figure 3.**
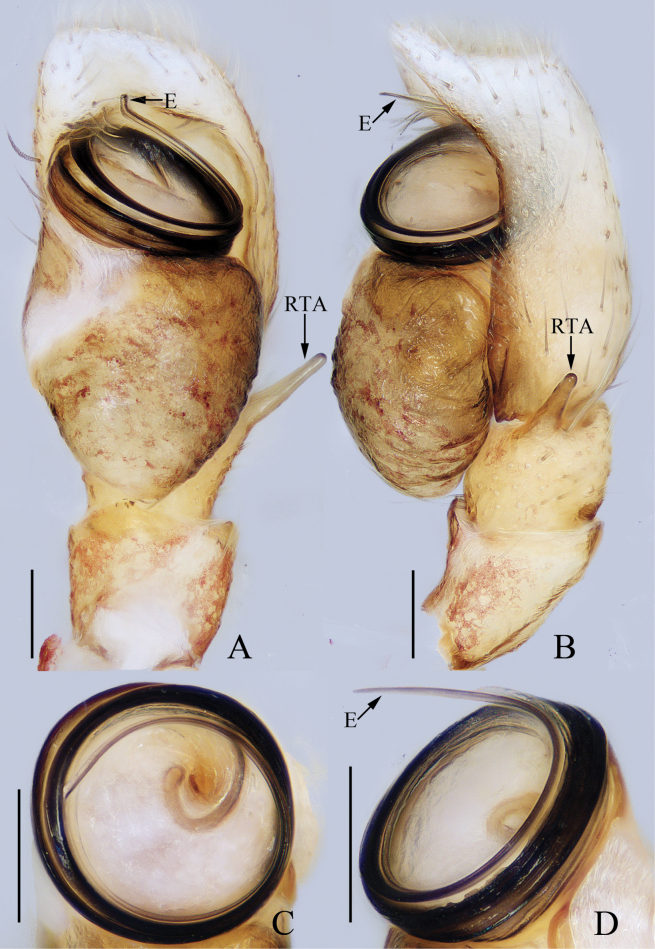
*Colaxescibagou* sp. nov., holotype (**A, B**) and male paratype (**C, D**) **A** palp, ventral **B** ditto, retrolateral **C** embolus, anteroventral **D** ditto, retrolateral. Scale bars: 0.1 mm.

**Figure 4.**
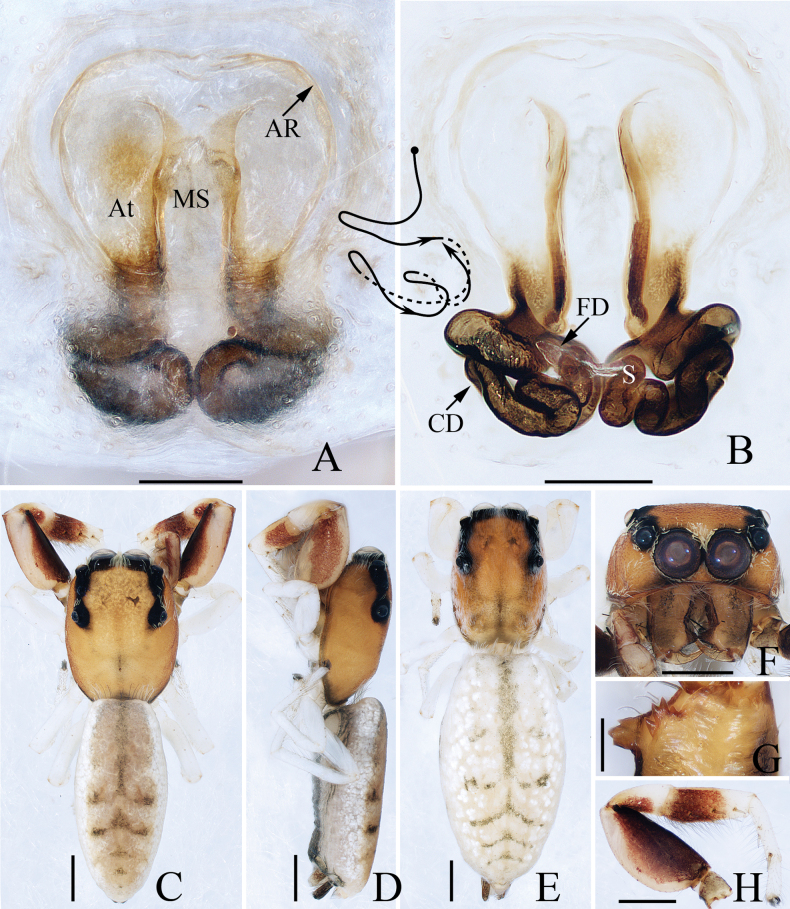
*Colaxescibagou* sp. nov., male holotype and female paratype **A** epigyne, ventral **B** vulva, dorsal **C** holotype habitus, dorsal **D** ditto, lateral **E** female paratype habitus, dorsal **F** holotype carapace, frontal **G** holotype chelicera, posterior **H** leg I of holotype, prolateral. Scale bars: 0.1 mm (**A, B, G**); 0.5 mm (**C–F, H**).

*Colaxescibagou* sp. nov. can be easily distinguished from other congeners by the wide embolic coils, which are equal to about four-fifths the bulb width in diameter, the male cheliceral promarginal fissidental tooth, and the presence of hood structure formed by the anterior portion of the epigynal median septum (Figs 3A, 4A, G), versus embolic coils less than two-thirds the bulb width in diameter, two or three male cheliceral promarginal teeth, and the absence of similar hood structure in *Colaxes* (for illustrations, see Metzner 2024).

#### Description.

**Male** (Figs 3, 4C, D, F–H). Total length 3.66. Carapace 1.55 long, 1.23 wide. Abdomen 2.21 long, 1.02 wide. Eye sizes and inter-distances: AME 0.34, ALE 0.17, PLE 0.13, AERW 0.96, PERW 1.04, EFL 0.62. ***Legs***: I 3.37 (1.08, 0.55, 0.83, 0.63, 0.28), II 2.28 (0.70, 0.38, 0.50, 0.45, 0.25), III 2.29 (0.70, 0.33, 0.50, 0.48, 0.28), IV 2.82 (0.88, 0.38, 0.63, 0.63, 0.30). Carapace sub-square, yellow except the eye bases black, without distinct markings, covered with pale thin setae on face and bilateral sides of eye field; fovea indistinct. Chelicerae dark yellow, each with promarginal fissidental tooth with two or three cusps, and two retromarginal teeth separated by fissidental tooth with two cusps. Endites paler than chelicerae and widened distally. Labium yellow to brown, the antero-submarginal portions pale. Sternum shield-shaped, covered with thin setae. Legs pale to red-brown; legs I strongest, with slightly inflated femora, and five and four ventral spines on tibiae and metatarsi, respectively. Abdomen elongated, dorsum pale to dark brown, with narrow, longitudinal, anteromedian, dark brown stripes followed by four chevron markings, covered with dense silver spots laterally, and prominent scutum extending across the whole surface; venter grey, with broad, green-brown, median longitudinal band.

***Palp*** (Fig. 3A–D): tibia slightly wider than long, with straight, antero-retrolaterally extended RTA tapered to blunt tip; cymbium about 1.5 times longer than wide; bulb swollen, slightly narrowed medio-posteriorly; embolus long, arising at the anterior portion of bulb, coiled more than twice, with blunt tip.

**Female** (Fig. 4A, B, E). Total length 4.51. Carapace 1.62 long, 1.17 wide. Abdomen 2.81 long, 1.55 wide. Eye sizes and inter-distances: AME 0.34, ALE 0.17, PLE 0.13, AERW 0.96, PERW 1.06, EFL 0.60. ***Legs***: I 2.62 (0.80, 0.48, 0.63, 0.43, 0.28), II 2.06 (0.58, 0.38, 0.50, 0.35, 0.25), III 2.14 (0.70, 0.33, 0.43, 0.43, 0.25), IV 2.77 (0.88, 0.33, 0.70, 0.58, 0.28). ***Habitus*** (Fig. 4E) similar to that of male except the less-developed legs I, the absence of abdomen dorsal scutum, and two cheliceral promarginal teeth.

***Epigyne and vulva*** (Fig. 4A, B): longer than wide; atrium oval, with invert U-shaped anterior ridge, and separated by broad median septum, which forms pair of hood structures at anterior portion; copulatory openings located at the lowest portions of atrium, slit-shaped, separated from each other about 1.5 times their width; copulatory ducts long, forming complicated coils; spermathecae indistinct; fertilization ducts lamellar, extending anterolaterally.

#### Distribution.

Known only from the type locality in Yunnan, China (Fig. 22B).

#### Comments.

The unpublished molecular evidence has supported the pairing.

### ﻿Genus Epeus

Taxon classificationAnimaliaAraneaeSalticidae

Peckham & Peckham, 1886

7C4FA0D7-1271-50AA-83D6-B9672D84096E

#### Type species.

*Evenustener* Simon, 1877.

#### Comments.

*Epeus*, one of the members of the subtribe Plexippina Simon, 1901 (Maddison 2015), contains 19 species distributed mainly in East, South, and Southeast Asia (WSC 2024). The genus has always been considered to be closely related to *Plexippoides* Prószyński, 1984 and a relatively comprehensive comparison of those two genera was provided by Logunov (2021), who summarized seven characters to distinguish *Epeus* and *Plexippoides*. However, the conclusion could not be perfect. Those two genera share similar palpal structure, especially in having a cluster of setae antero-retrolateral to the bulb on cavity, the presence of tegular lobe, and the sclerotized RCA; however, *Epeus* can be distinguished from *Plexippoides* by the following: 1) the slender body, covered with sparse setae on carapace (for illustrations, see Metzner 2024), versus rather dumpy body, setose on carapace in *Plexippoides* (Logunov 2021: figs 1, 6, 9, 14, 45, 50); 2) the most anterior margin of bulb cavity is far away from cymbial tip at least ca. one-third the cymbial length (for illustrations, see Metzner 2024), versus close to cymbial tip no more than one-third the cymbial length in *Plexippoides* (Lougunov 2021: figs 17, 23, 28); 3) the weakly sclerotized copulatory ducts run posteriorly and form multi-loops (Patoleta et al. 2020), but sclerotized copulatory ducts do not form similar loops in *Plexippoides* (Logunov 2021: figs 33, 37, 41). *P.guangxi* and *P.dilucidus* have slender bodies, and their most anterior margin of bulb cavity is far away from cymbial tip more than one-third the cymbial length (Peng and Li 2002: fig. 3A, C; Próchniewicz 1990: figs 22, 23, 26). Based on that, they are being transferred.

### ﻿ Epeus pengi sp. nov.

Taxon classificationAnimaliaAraneaeSalticidae

A8FD1DAE-54DC-5122-9CA5-608CDB1BFCF4

https://zoobank.org/812997F9-7D51-426E-938B-C0548CC3E819

Figs 5 , 6 , 22B

#### Type material.

***Holotype*** ♂ (TRU-XZ-JS-0010), China: Xizang: Bowo County, 318 National Highway, nearby the 102 Tunnel (30°4.41′N, 95°7.99′E, ca 2160 m), 30 Jun. 2023, C. Wang leg. ***Paratypes*** 1♀ (TRU-XZ-JS-0011), same data as for holotype; 3♀ (TRU-XZ-JS-0012–0014), Zayu County, Cibagou National Nature Reserve (28°34.07′N, 97°5.44′E, ca 1620 m), 22 Jun. 2023, C. Wang leg.

#### Etymology.

The species name is a patronym in honour of Prof. Xianjin Peng, who has significantly contributed to the taxonomy of Chinese salticids; noun (name) in genitive case.

#### Diagnosis.

**Figure 5.**
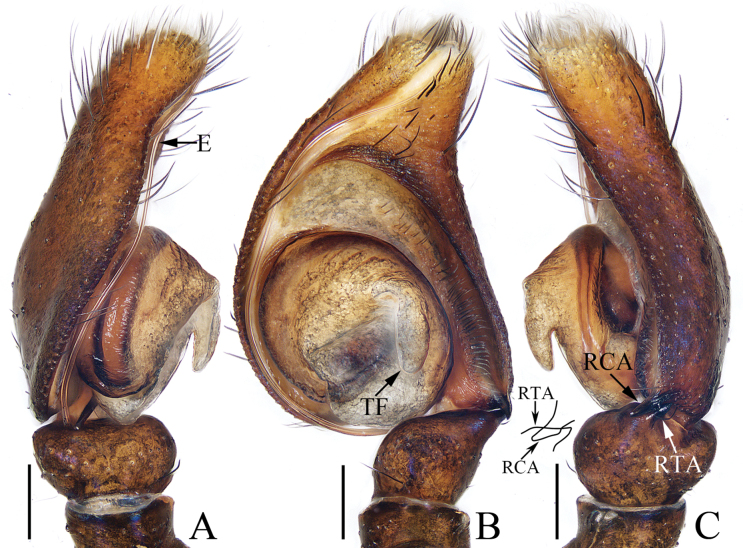
Male palp of *Epeuspengi* sp. nov., holotype **A** prolateral **B** ventral **C** retrolateral. Scale bars: 0.2 mm.

**Figure 6.**
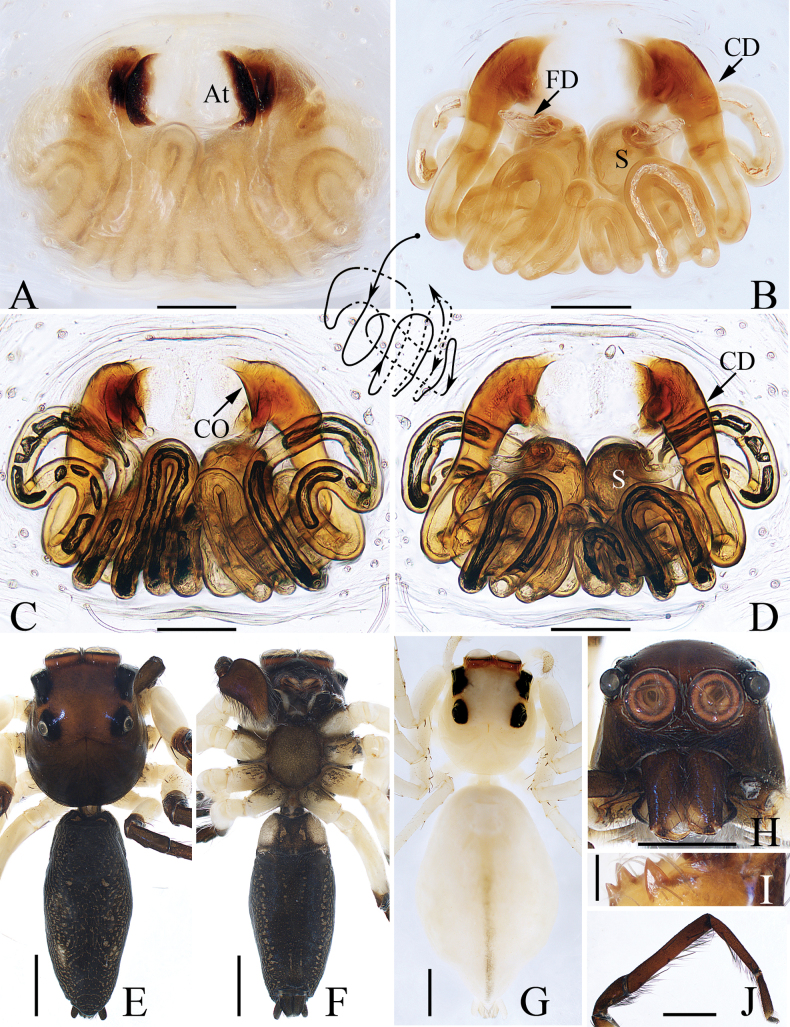
*Epeuspengi* sp. nov., male holotype and female paratype **A, C** epigyne, ventral **B, D** vulva, dorsal **E** holotype habitus, dorsal **F** ditto, ventral **G** female paratype habitus, dorsal **H** holotype carapace, frontal **I** holotype chelicera, posterior **J** leg I of holotype, prolateral. Scale bars: 0.1 mm (**A–D, I**); 1.0 mm (**E–H, J**).

The male of *Epeuspengi* sp. nov. closely resembles that of *E.dilucidus* (Próchniewicz, 1990) comb. nov. in the general shape of palp, but it can be distinguished as follows: 1) RTA crossed with RCA in ventral view (Fig. 5B), versus not crossed in *E.dilucidus* (Próchniewicz 1990: figs 21, 25); 2) RCA only slightly curved in ventral view (Fig. 5B), versus curved about 90° in *E.dilucidus* (Próchniewicz 1990: figs 21, 25). The male also somewhat resembles that of *E.guangxi* (Peng & Li, 2002), comb. nov. in having a similar palp, but can be easily distinguished by the lack of brushes on the femora and tibiae of legs I (Fig. 6J), versus the presence of brushes formed by greyish-black long bristles ventrally and dorsally on the tibiae and metataisi of legs I (see the description of Peng and Li 2002). The female is almost indistinguishable from *E.bicuspidatus* (Song, Gu & Chen, 1988) both in habitus and copulatory organs, but can be distinguished by the thicker copulatory ducts, which do not extend beyond the copulatory openings (Fig. 6A–D), versus thinner copulatory ducts, extending beyond the copulatory openings in *E.bicuspidatus* (Meng et al. 2015: figs 11–14).

#### Description.

**Male** (Figs 5, 6E, F, H–J). Total length 5.84. Carapace 2.35 long, 2.00 wide. Abdomen 3.39 long, 1.45 wide. Eye sizes and inter-distances: AME 0.60, ALE 0.30, PLE 0.27, AERW 1.74, PERW 1.61, EFL 1.16. ***Legs***: I 7.90 (2.35, 1.00, 2.20, 1.50, 0.85), II 7.70 (2.35, 1.00, 2.00, 1.50, 0.85), III 8.70 (2.65, 1.00, 2.00, 2.05, 1.00), IV 8.50 (2.40, 0.85, 2.00, 2.25, 1.00). Carapace orange-brown to dark, with sub-square, elevated cephalon, and sloped thorax with half round margin; fovea dark red, longitudinal. Chelicerae red-brown, each with two promarginal teeth and one retromarginal tooth. Endites sub-square, with pale inner portions. Labium near linguiform, with pale anterior portion. Sternum dark brown, mingled with green, slightly longer than wide, legs pale to red-brown. Abdomen elongated, dorsum dark brown, spotted; venter coloured as dorsum.

***Palp*** (Fig. 5A–C): tibia swollen in retrolateral view, with short, strongly sclerotized RTA widened at base, and with pointed tip directed towards about 1: 30 o’clock position in retrolateral view; cymbium about 1.5 times longer than wide in ventral view, bearing cluster of dark setae retrolateral to the bulb on cavity, and with base-retrolateral apophysis tapered to relatively pointed tip; bulb swollen, almost round, with posteriorly extended tegular flap originating at the antero-retrolateral submargin; embolus originating at ca 5 o’clock position of bulb, flagelliform, extending ca half circle along the bulb and then antero-retrolaterally extending to the cymbial tip.

**Female** (Fig. 6A–D, G). Total length 7.17. Carapace 2.21 long, 2.08 wide. Abdomen 4.58 long, 3.04 wide. Eye sizes and inter-distances: AME 0.58, ALE 0.25, PLE 0.21, AERW 1.73, PERW 1.58, EFL 1.17. ***Legs***: I 6.90 (2.00, 1.00, 1.85, 1.30, 0.75), II 6.90 (2.15, 1.00, 1.75, 1.25, 0.75), III 7.95 (2.50, 1.00, 1.75, 1.85, 0.85), IV 7.80 (2.25, 0.85, 1.85, 2.00, 0.85). Carapace and abdomen (Fig. 6G) pale yellow; dorsum of abdomen with narrow, longitudinal stripe extending antero-medially to the terminus.

***Epigyne and vulva*** (Fig. 6A–D): wider than long, with anterior, oval atrium; copulatory openings anterolaterally located, with C-shaped margins; copulatory ducts long, slightly widened at base, forming complicated coils; spermathecae almost spherical, touched, medially located; fertilization ducts originating from anterior portions of spermathecae, extending transversely.

#### Distribution.

Known only from the type locality in Xizang, China (Fig. 22B).

### ﻿Genus Evarcha

Taxon classificationAnimaliaAraneaeSalticidae

Simon, 1902

12CD7BD3-041E-5DCA-B5EB-B6553EC9289A

#### Type species.

*Araneusfalcatus* Clerck, 1757.

#### Comments.

*Evarcha*, one of the largest genera of the subtribe Plexippina Simon, 1901 (Maddison 2015), contains 92 worldwide distributed species (WSC 2024). The genus has a vast diversity in genital morphology: embolus ranging from short, stout and compact to very long and filamentous, tegulum ranging from rounded to more complex shapes bearing outgrowths, insemination ducts ranging from broad and membranous to thin and tube-shaped might indicate it is more of a ‘hold all’ genus harbouring unrelated species (Kanesharatnam and Benjamin 2021). Based on the above, a valid definition of the genus could not be proposed. We assigned the new species to the genus because it shares copulatory organs similar to some known species, such as *E.laetabunda* (C. L. Koch, 1846) and *E.michailovi* Logunov, 1992.

### ﻿ Evarcha zayu sp. nov.

Taxon classificationAnimaliaAraneaeSalticidae

C0FD27F3-77BF-533C-8546-4D7086F7E114

https://zoobank.org/FE6BE80E-C2DD-41A2-8927-FEAD4441BF44

Figs 7 , 8 , 22A

#### Type material.

***Holotype*** ♂ (IZCAS-Ar44770), China: Xizang: Zayu County, Zhuwagen Township (28°40.27′N, 97°27.15′E, ca 2330 m), 18–19 Jul. 1994, S. Wu leg. ***Paratypes*** 3♀ (IZCAS-Ar44771–773), same data as for holotype.

#### Etymology.

The species name is a noun derived from the type locality: Zayu County.

#### Diagnosis.

**Figure 7.**
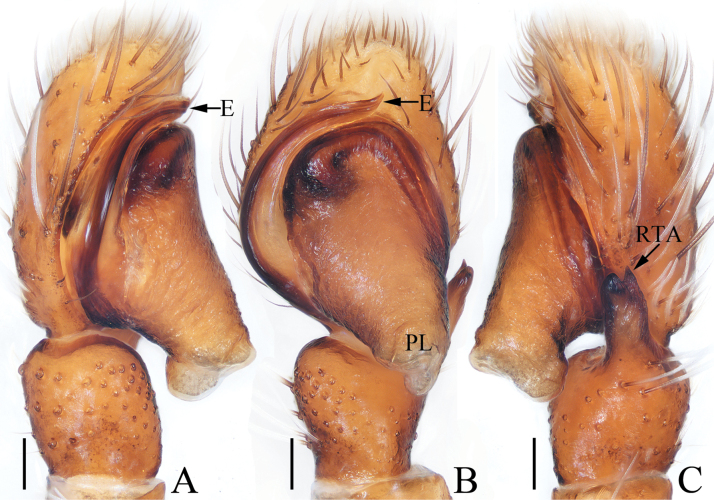
Male palp of *Evarchazayu* sp. nov., holotype **A** prolateral **B** ventral **C** retrolateral. Scale bars: 0.1 mm.

**Figure 8.**
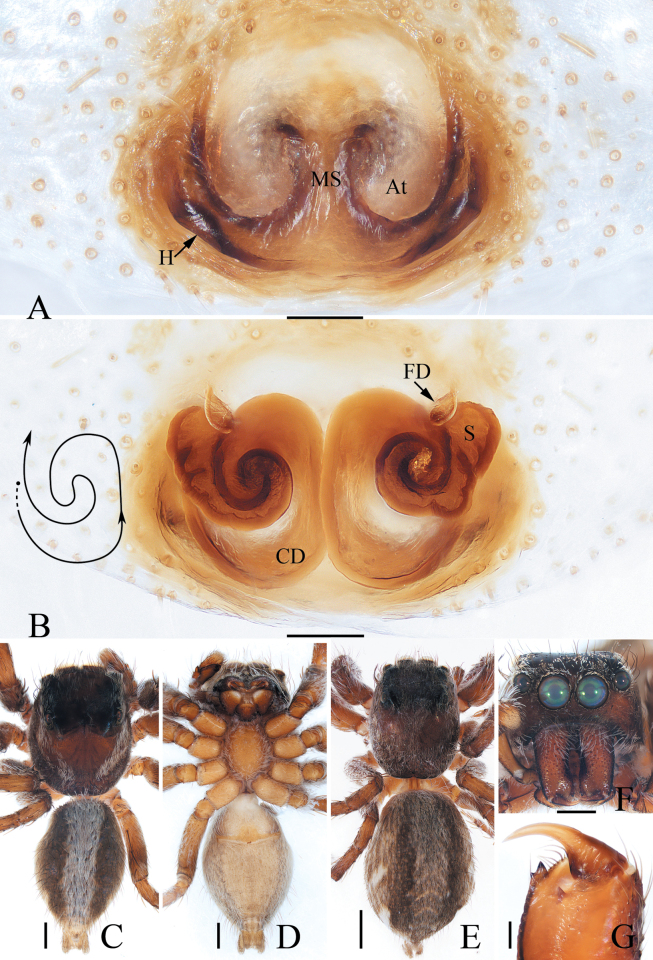
*Evarchazayu* sp. nov., male holotype and female paratype **A** epigyne, ventral **B** vulva, dorsal **C** holotype habitus, dorsal **D** ditto, ventral **E** female paratype habitus, dorsal **F** holotype carapace, frontal **G** holotype chelicera, posterior. Scale bars: 0.1 mm (**A, B, G**); 0.5 mm (**C, D, F**); 1.0 mm (**E**).

*Evarchazayu* sp. nov. closely resembles *E.laetabunda* (C. L. Koch, 1846) in having very similar copulatory organs, but can be distinguished as follows: 1) RTA bifurcated with two rami in retrolateral view (Fig. 7C), versus not bifurcated in *E.laetabunda* (Logunov 1992: fig. 2B); 2) median septum almost square (Fig. 8A), versus almost triangular in *E.laetabunda* (Logunov 1992: fig. 3A). The species also somewhat resembles *E.michailovi* Logunov, 1992 in the general shape of copulatory organs, but it differs as follows: 1) RTA bifurcated with two rami in retrolateral view (Fig. 7C), versus not bifurcated in *E.michailovi* (Logunov 1992: fig. 2D); 2) median septum about half the atrial length (Fig. 8A), versus less than one-third the atrial length in *E.michailovi* (Logunov 1992: fig. 3C).

#### Description.

**Male** (Figs 7, 8C, D, F, G). Total length 5.03. Carapace 2.35 long, 2.00 wide. Abdomen 2.62 long, 1.71 wide. Eye sizes and inter-distances: AME 0.44, ALE 0.26, PLE 0.25, AERW 1.62, PERW 1.50, EFL 1.00. ***Legs***: I 5.84 (1.63, 1.08, 1.48, 1.00, 0.65), II 4.41 (1.33, 0.83, 1.00, 0.75, 0.50), III 5.62 (1.83, 0.95, 1.13, 1.08, 0.63), IV 4.88 (1.50, 0.65, 1.00, 1.10, 0.63). Carapace red-brown to dark brown, covered with dense white and dark setae; fovea longitudinal, dark. Chelicerae red-brown, each with two promarginal teeth and one retromarginal tooth, with small dark tubercle bearing pale scale-like setae on base half of anterior surface. Endites slightly longer than wide, and widened distally, with pale distal inner portions. Labium almost linguiform. Sternum somewhat longer than wide, with straight anterior margin, covered with dense pale setae sub-marginally. Legs yellow to dark brown, setose. Abdomen sub-oval, dorsum pale to brown, covered with sparse, dark, long setae laterally, and broad, central, longitudinal, pale setal band about 1/3 the abdominal width, extending across the surface; venter pale grey.

***Palp*** (Fig. 7A–C): tibia slightly wider than long in retrolateral view, with straight, anteriorly extended RTA bifurcated distally into sclerotized ventral ramus and apically pointed dorsal ramus; cymbium longer than wide, covered with long setae; bulb swollen medio-posteriorly, with well-developed posterior lobe extending postero-dorsally at terminus; embolus broad, originating at ca 6 o’clock position of bulb, curved ca half circle along the prolateral side of bulb, with notably pointed tip.

**Female** (Fig. 8A, B, E). Total length 7.28. Carapace 3.00 long, 2.36 wide. Abdomen 4.04 long, 2.84 wide. Eye sizes and inter-distances: AME 0.52, ALE 0.28, PLE 0.24, AERW 1.72, PERW 1.72, EFL 1.12. ***Legs***: I 5.71 (1.70, 1.20, 1.33, 0.85, 0.63), II 5.66 (1.75, 1.20, 1.25, 0.83, 0.63), III 7.11 (2.38, 1.25, 1.38, 1.35, 0.75), IV 6.61 (2.00, 1.00, 1.38, 1.40, 0.83). ***Habitus*** (Fig. 8E) similar to that of male except darker in colour and without white setae on carapace.

***Epigyne and vulva*** (Fig. 8A, B): slightly wider than long, with pair of posterolateral hoods; atrium big, almost oval, separated by sub-square median septum about half the atrial length; copulatory ducts long, curved into U-shape anteromedially, and forming coils distally; spermathecae elongated; fertilization ducts extending anterolaterally.

#### Distribution.

Known only from the type locality in Xizang, China (Fig. 22A).

### ﻿Genus Icius

Taxon classificationAnimaliaAraneaeSalticidae

Simon, 1876

15294DD7-BD26-5431-87FE-F45631B68429

#### Type species.

*Icelusnotabilis* C. L. Koch, 1846.

#### Comments.

*Icius*, belongs to the tribe Chrysillini Simon, 1901, and is represented by 38 nominal species widely distributed from all over the world (WSC 2024). Like *Evarcha*, the genus might be more of a ‘hold all’ genus harbouring unrelated species. We assigned the below new species to the genus because it shares very similar habitus and relatively consistent copulatory organs with the generotype, *I.hamatus* (C. L. Koch, 1846). The generic position of the new species needs further attention. It is worth mentioning that the specimens described as *Iciushamatus* and *Phintellaversicolor* in Hu (2001) are misidentified (the misidentification of *Iciushamatus* was documented by WSC 2024), and they are most closely related to *Iciuszang* sp. nov. morphologically.

### ﻿ Icius zang sp. nov.

Taxon classificationAnimaliaAraneaeSalticidae

DB5795EF-0380-5D61-9792-AB8B8568D721

https://zoobank.org/B0386D54-4320-432A-B7E4-DDC694A01EEC

Figs 9 , 10 , 22A

#### Type material.

***Holotype*** ♂ (IZCAS-Ar44774), China: Xizang: Lhasa City (29°39.11′N, 91°6.78′E, ca 3660 m), 26 Aug. 2001, X. Peng leg. ***Paratypes*** 3♂3♀ (IZCAS-Ar44775–44780), same data as for holotype.

#### Etymology.

The specific name is after one of the most popular minorities (Zang) in Xizang, China; noun.

#### Diagnosis.

**Figure 9.**
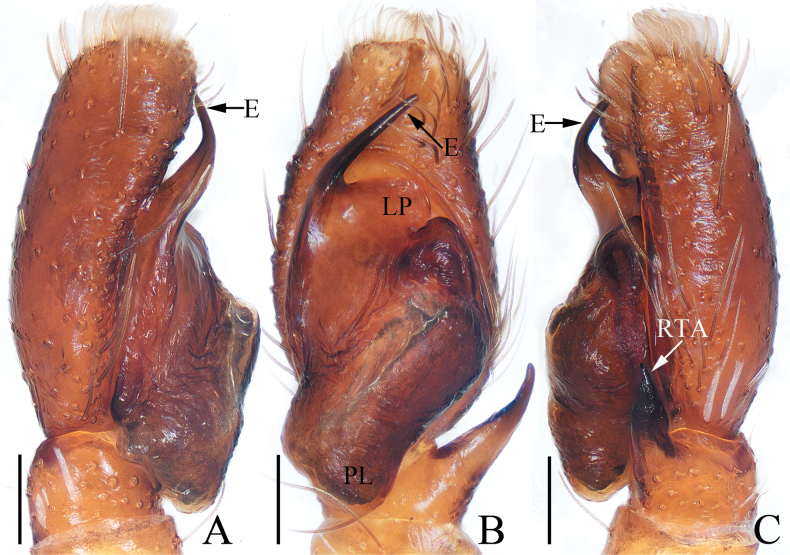
Male palp of *Iciuszang* sp. nov., holotype **A** prolateral **B** ventral **C** retrolateral. Scale bars: 0.1 mm.

**Figure 10.**
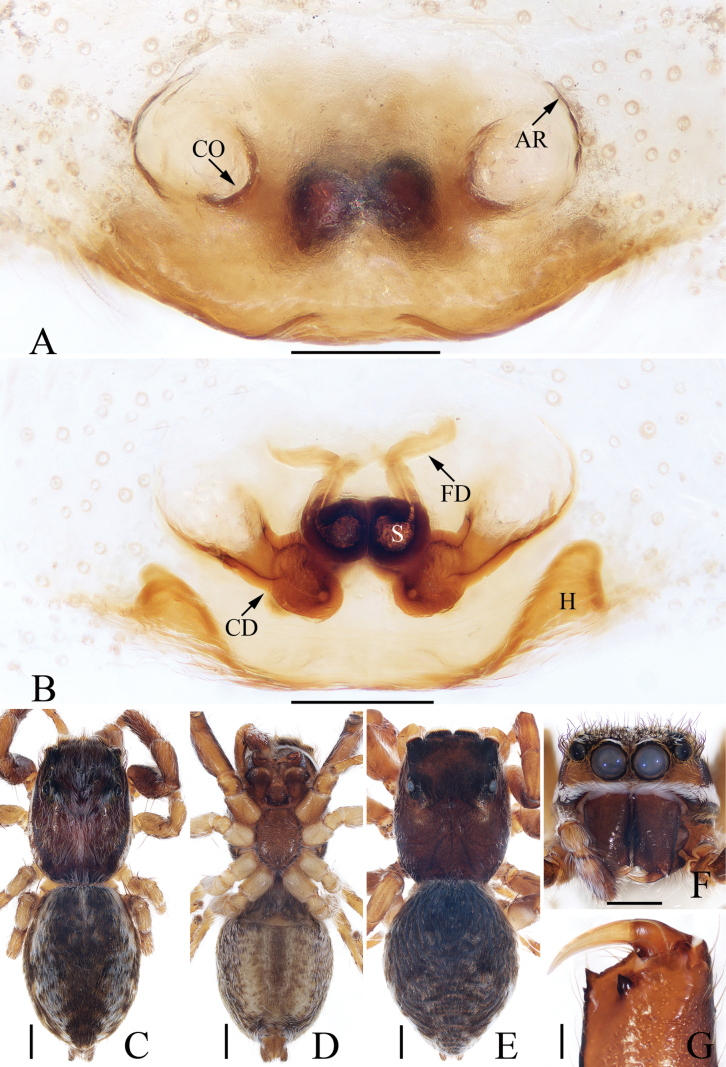
*Iciuszang* sp. nov., male holotype and female paratype **A** epigyne, ventral **B** vulva, dorsal **C** holotype habitus, dorsal **D** ditto, ventral **E** female paratype habitus, dorsal **F** holotype carapace, frontal **G** holotype chelicera, posterior. Scale bars: 0.1 mm (**A, B, G**); 0.5 mm (**C–F**).

*Iciuszang* sp. nov. resembles that of *I.hamatus* (C. L. Koch, 1846) in having similar habitus and the general shape of copulatory organs but can be easily distinguished by the presence of only one tibial apophysis and the pair of epigynal hoods (Figs 9B, C, 10A, B) versus two tibial apophyses and only one epigynal hood in *I.hamatus* (Peng 2020: fig. 123a–d).

#### Description.

**Male** (Figs 9, 10C, D, F, G). Total length 4.53. Carapace 2.05 long, 1.55 wide. Abdomen 2.40 long, 1.75 wide. Eye sizes and inter-distances: AME 0.36, ALE 0.19, PLE 0.19, AERW 1.13, PERW 1.18, EFL 0.80. ***Legs***: I 4.15 (1.30, 0.75, 1.00, 0.70, 0.40), II 3.04 (0.90, 0.55, 0.68, 0.53, 0.38), III 3.06 (0.90, 0.50, 0.68, 0.60, 0.38), IV 3.65 (1.13, 0.53, 0.78, 0.78, 0.43). Carapace red-brown, setose, with scale-like setal bands on anterior and lateral margins; fovea longitudinal. Chelicerae red-brown, each with two promarginal teeth and one retromarginal tooth. Endites longer than wide, widened distally. Labium almost linguiform. Sternum ca 1.5 times longer than wide, covered with long pale thin setae. Legs yellow to red-brown, setose. Abdomen oval, dorsum dark brown, with pair of longitudinal, setal pale stripes laterally; venter pale brown to brown.

***Palp*** (Fig. 9A–C): tibia wider than long; RTA slightly broadened anteromedially and slightly curved towards bulb distally, with pointed tip; cymbium ca two times as long as wide; bulb swollen medio-posteriorly, with blunt posterior lobe; embolus originating at antero-prolateral portion of bulb, curved medially and blunt apically, accompanied by half-round lamellar process near the base.

**Female** (Fig. 10A, B, E). Total length 4.82. Carapace 2.36 long, 1.77 wide. Abdomen 2.67 long, 2.05 wide. Eye sizes and inter-distances: AME 0.41, ALE 0.26 PLE 0.19, AERW 1.41, PERW 1.36, EFL 0.85. ***Legs***: I 4.11 (1.25, 0.83, 0.95, 0.63, 0.45), II 3.79 (1.18, 0.75, 0.83, 0.58, 0.45), III 3.61 (1.15, 0.63, 0.70, 0.70, 0.43), IV 5.48 (1.75, 0.78, 1.35, 1.05, 0.55). ***Habitus*** (Fig. 10E) similar to that of male but without dense setae, white scale-like setal marginal bands on carapace, and pair of longitudinal, pale setal bands on abdomen.

***Epigyne and vulva*** (Fig. 10A, B): wider than long, with pair of posterolateral hoods; atrium large, anterior located, with C-shaped lateral ridges; copulatory openings almost half round, open laterally; copulatory ducts thick, strongly curved medially; spermathecae almost spherical, touching; fertilization ducts originating from the lateral-anterior portion of spermathecae.

#### Distribution.

Known only from the type locality in Xizang, China (Fig. 22A).

### ﻿Genus Pancorius

Taxon classificationAnimaliaAraneaeSalticidae

Simon, 1902

1B5C9F87-3436-5015-A334-DEF0ECDF6EB9

#### Type species.

*Erganedentichelis* Simon, 1899.

#### Comments.

*Pancorius*, contains 45 species mainly distributed in East, South, and Southeast Asia (WSC 2024). The genus is distinguishable from closely related genera *Colopsus* Simon, 1902, *Evarcha*, *Hyllus* C. L. Koch, 1846 by sandy brown habitus with pale white central and lateral carapace bands, serrated longitudinal abdominal band, simple palp with rounded or oval bulb, short embolus, single RTA with pointed tip, epigyne with sizeable central pocket, comparably small membranous window and multi-chambered spermathecae (Kanesharatnam and Benjamin 2021). The below-described new species is placed in the genus because it generally resembles that of most species. However, it is worth mentioning that it is specific for having a very long embolus originating from the median portion of the bulb’s prolateral side and with a membranous portion at base that may indicate its generic position needs further attention.

### ﻿ Pancorius nyingchi sp. nov.

Taxon classificationAnimaliaAraneaeSalticidae

E78CB722-E09F-5EB6-B044-D9B4BA2CD64E

https://zoobank.org/4AE822ED-CEB6-4956-B132-D8DBFB7466AA

Figs 11 , 12 , 22A

#### Type material.

***Holotype*** ♂ (TRU-XZ-JS-0015), China: Xizang: Zayu County, Cibagou National Nature Reserve (28°41.43′N, 97°2.86′E, ca 2570 m), 23 Jun. 2023, C. Wang leg. ***Paratypes*** 8♂10♀ (TRU-XZ-JS-0016–0033), same data as for holotype; 5♂3♀ (TRU-XZ-JS-0034–0041), Bowo County, 318 National Highway, nearby the 102 Tunnel (30°4.41′N, 95°7.99′E, ca 2160 m), 30 Jun. 2023, C. Wang leg.; 5♂3♀ (TRU-XZ-JS-0042–0049), Medog County, Renqingbengsi Scenic Area (29°18.10′N, 95°21.29′E, ca 2040), 18 Aug. 2023, C. Wang and H. Yao leg.

#### Etymology.

The species name is a noun in apposition derived from Nyingchi City. The type localities Zayu, Bowo, Medog belong to the municipal administration of Nyingchi.

#### Diagnosis.

**Figure 11.**
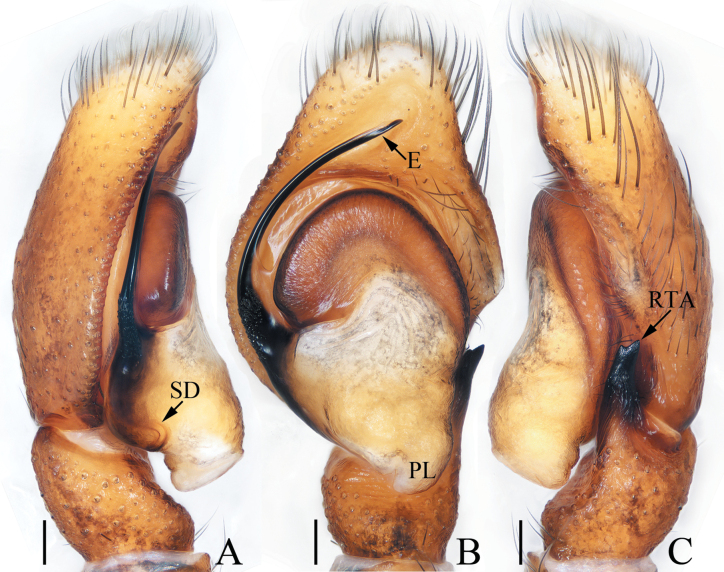
Male palp of *Pancoriusnyingchi* sp. nov., holotype **A** prolateral **B** ventral **C** retrolateral. Scale bars: 0.1 mm.

**Figure 12.**
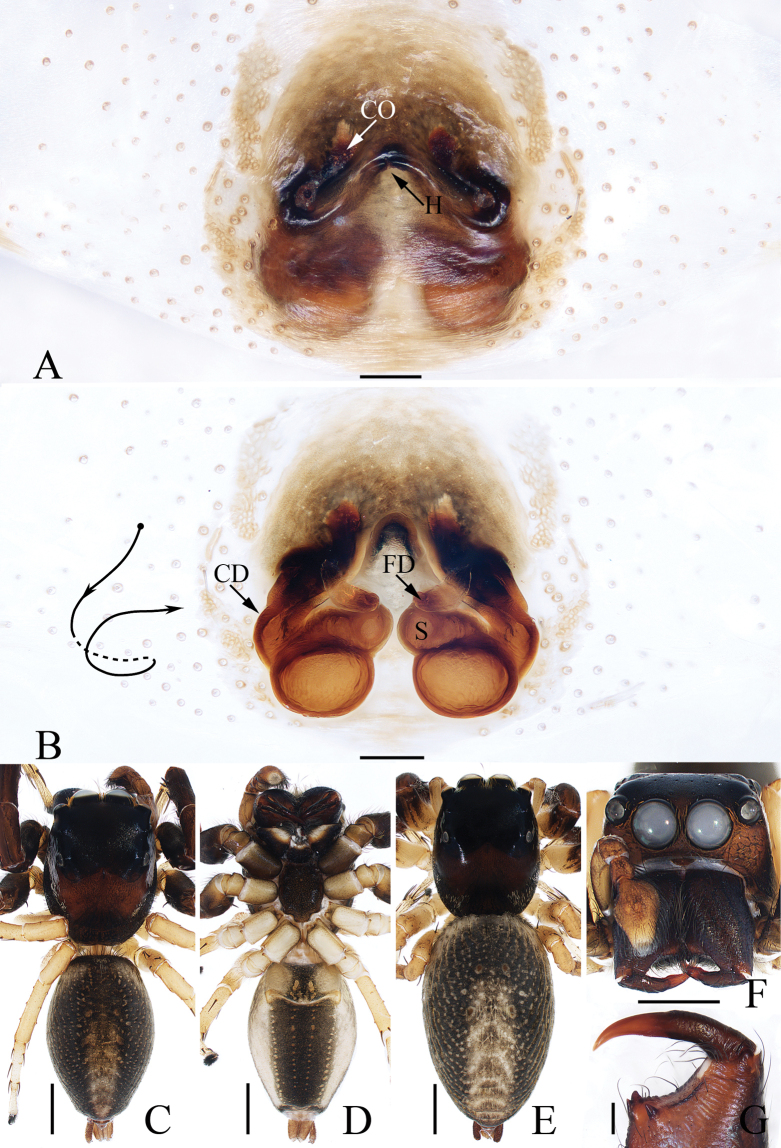
*Pancoriusnyingchi* sp. nov., male holotype and female paratype **A** epigyne, ventral **B** vulva, dorsal **C** holotype habitus, dorsal **D** ditto, ventral **E** female paratype habitus, dorsal **F** holotype carapace, frontal **G** holotype chelicera, posterior. Scale bars: 0.1 mm (**A, B**); 0.2 (**G**); 1.0 mm (**C–F**).

*Pancoriusnyingchi* sp. nov. resembles *P.manipuriensis* (Biswas & Biswas, 2004) in the general shape of copulatory organs, but it can be easily distinguished as follows: 1) embolus originating at ca 9:00 o’clock position of bulb (Fig. 11A, B), versus ca 10:30 o’clock position of bulb in *P.manipuriensis* (Caleb 2023: fig. 15); 2) embolus is almost as long as bulb (Fig. 11B), versus less than half the bulb length in *P.manipuriensis* (Caleb 2023: fig. 15); 3) epigyne with a single hood (Fig. 12A), versus a pair of hoods in *P.manipuriensis* (Caleb 2023: figs 21, 22)

#### Description.

**Male** (Figs 11, 12C, D, F, G). Total length 6.30. Carapace 2.83 long, 2.26 wide. Abdomen 3.30 long, 2.17 wide. Eye sizes and inter-distances: AME 0.62, ALE 0.38, PLE 0.33, AERW 1.96, PERW 1.91, EFL 1.17. ***Legs***: I 7.30 (2.05, 1.30, 1.90, 1.30, 0.75), II 5.70 (1.75, 1.15, 1.30, 0.90, 0.60), III 6.20 (2.00, 1.00, 1.25, 1.25, 0.70), IV 6.35 (1.90, 1.00, 1.40, 1.40, 0.65). Carapace red-brown to dark brown, with two clusters of lateral, white setae, and big, red-brown area on thorax; fovea longitudinal, dark. Chelicerae red-brown, each with two promarginal teeth and one retromarginal tooth. Endites dark brown, with pale inner-distal portions. Labium coloured same as endites, with pale anterior margin bearing several dark setae. Sternum dark brown, longer than wide, with straight anterior margin. Legs yellow to dark brown, with three and two pairs of ventral spines on tibiae and metatarsi I, respectively. Abdomen elongated, dorsum dark brown, dotted, with pair of anterolateral pale stripes, two pairs of median muscle depressions, and longitudinal, central pale band extending from middle to the terminus; venter pale bilaterally, with broad, dark brown band bearing two pairs of longitudinal, dotted lines.

***Palp*** (Fig. 11A–C): tibia wider than long, with strongly sclerotized RTA almost equal in length with tibia, bifurcated with two small sub-triangular rami distally; cymbium about 1.5 times as long as wide; bulb flat, with posterior lobe extending postero-prolaterally; embolus strongly sclerotized, widened at base, curved around the prolateral margin of bulb into C-shape, with noticeably pointed tip.

**Female** (Fig. 12A, B, E). Total length 6.84. Carapace 2.71 long, 2.22 wide. Abdomen 4.31 long, 2.67 wide. Eye sizes and inter-distances: AME 0.62, ALE 0.36, PLE 0.31, AERW 1.87, PERW 1.87, EFL 1.11. ***Legs***: I 5.05 (1.50, 1.00, 1.20, 0.75, 0.60), II 4.85 (1.50, 1.00, 1.05, 0.70, 0.60), III 5.80 (1.90, 0.95, 1.20, 1.10, 0.65), IV 6.00 (1.90, 0.95, 1.30, 1.20, 0.65). ***Habitus*** (Fig. 12E) similar to that of male.

***Epigyne and vulva*** (Fig. 12A, B): longer than wide, with downward opened, antero-central hood; copulatory openings slit-shaped, anterolaterally located; copulatory ducts curved and twisted; spermathecae indistinct; fertilization ducts lamellar, anterolaterally extending.

#### Distribution.

Known only from the type locality in Xizang, China (Fig. 22A).

### ﻿Genus Phintella

Taxon classificationAnimaliaAraneaeSalticidae

Strand, 1906

98473FA0-50D0-521E-A48A-EB0AEA384F5A

#### Type species.

*Phintellatypica* Strand, 1906.

#### Comments.

**Figure 13.**
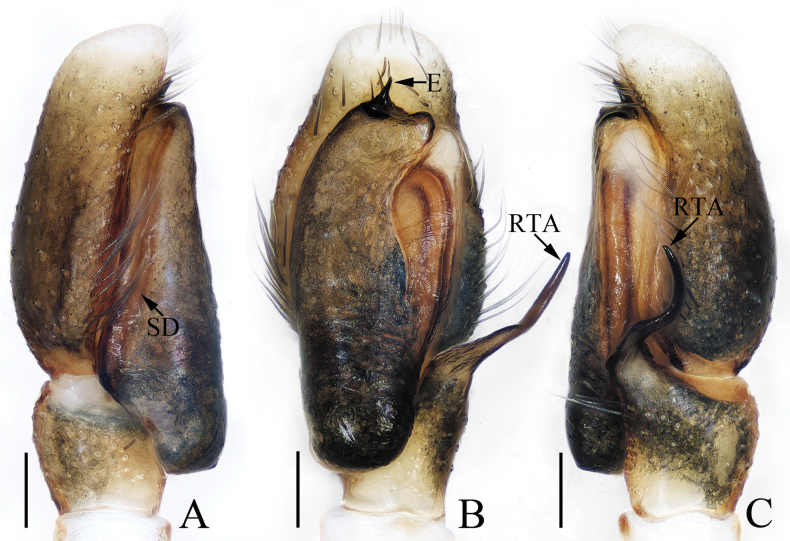
Male palp of *Phintellalongapophysis* Lei & Peng, 2013 **A** prolateral **B** ventral **C** retrolateral. Scale bars: 0.1 mm.

**Figure 14.**
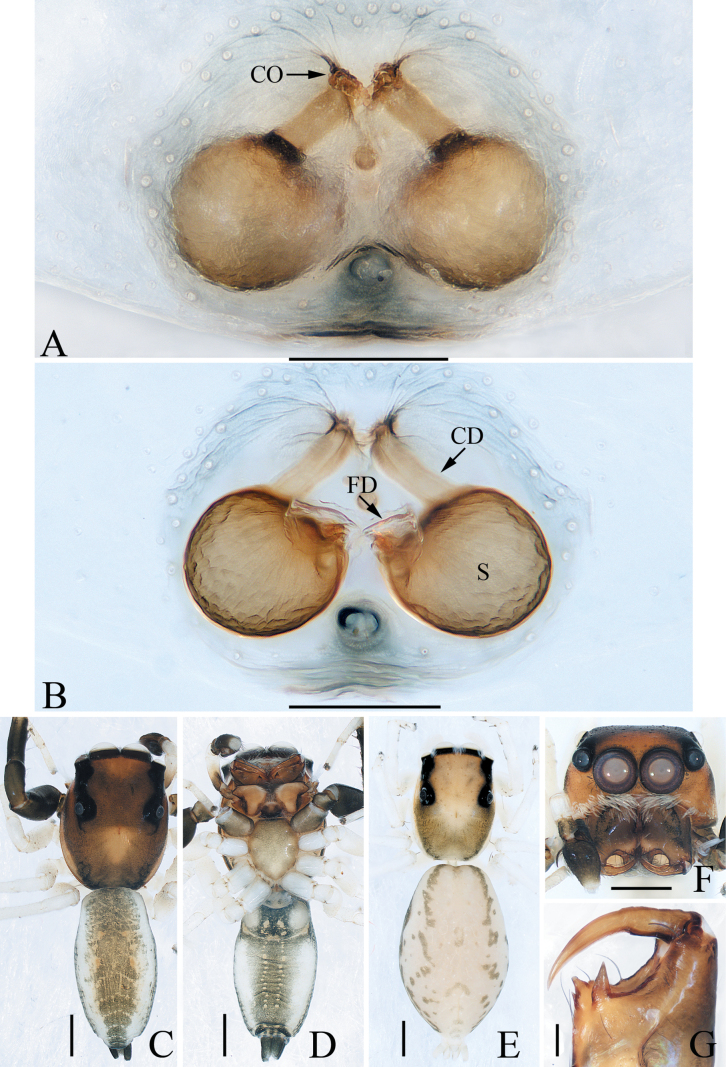
*Phintellalongapophysis* Lei & Peng, 2013 **A** epigyne, ventral **B** vulva, dorsal **C** male habitus, dorsal **D** ditto, ventral **E** female habitus, dorsal **F** male carapace, frontal **G** male chelicera, posterior. Scale bars: 0.1 mm (**A, B, G**); 0.5 mm (**C–F**).

*Phintella*, one of the species-richest genera of the tribe Chrysillini Simon, 1901, contains 72 species mainly distributed in Asia and Africa (WSC 2024). The genus is diverse in habitus and copulatory organs, which indicates it should be split or at least should be further divided into groups. *Phintellalongapophysis* is a convincing sample. It is sexual dimorphism in habitus, with hook-shaped distal apophysis on endites and distal-retrolateral tegular lobe (Figs 13B, 14C–E) that are different from the generotype and its congeners, which without sexual dimorphism, lacks the hook-shaped distal apophysis on endites, and with the lamellar process instead of distal-retrolateral tegular lobe (for illustration, see Metzner 2024).

Moreover, *Phintellasufflava* (Jastrzębski, 2009), comb. nov. is transferred because it shares a similar epigyne and vulva with *P.longapophysis* Lei & Peng, 2013. In the following, we considered *P.longapophysis* Lei & Peng, 2013 as a valid species rather than a synonym of the latter because the examined female specimens consistently have spherical spermathecae, which is different from *P.sufflava*. However, we cannot confirm whether the difference is due to interspecific differences or intraspecific variations. So, the status of the two mentioned species needs further confirmation.

### ﻿ Phintella longapophysis

Taxon classificationAnimaliaAraneaeSalticidae

Lei & Peng, 2013

F2A608CA-87EB-5544-8918-C8D3CB34FCC2

Figs 13 , 14 , 22A

Phintella longapophysis Lei & Peng, 2013: 100, figs 1, 2a–c (male holotype, not examined).

#### Material examined.

7♂9♀ (TRU-XZ-JS-0050–0065), China: Xizang: Zayu County, Cibagou National Nature Reserve (28°41.43′N, 97°2.86′E, ca 2570 m), 23 Jun. 2023, C. Wang leg.; 3♂2♀ (TRU-XZ-JS-0066–0070), Bowo County, 318 National Highway, nearby the 102 Tunnel (30°4.41′N, 95°7.99′E, ca 2160 m), 30 Jun. 2023, C. Wang leg.

#### Diagnosis.

The male was thoroughly diagnosed by Lei and Peng (2013). The female of this species closely resembles that of *P.sufflava* (Jastrzębski, 2009), comb. nov. in having very similar epigyne and vulva, but it can be distinguished by the spherical spermathecae (Fig. 14B) versus sub-square spermathecae in *P.sufflava* (Jastrzębski 2009: fig. 4).

#### Description.

**Male** (Figs 13, 14C, D, F, G). See Lei and Peng (2013).

**Female** (Fig. 14A, B, E). Total length 4.06. Carapace 1.56 long, 1.10 wide. Abdomen 2.48 long, 1.24 wide. Eye sizes and inter-distances: AME 0.36, ALE 0.20, PLE 0.19, AERW 1.04, PERW 1.08, EFL 0.84. ***Legs***: I 2.63 (0.75, 0.50, 0.63, 0.45, 0.30), II 2.40 (0.75, 0.40, 0.55, 0.40, 0.30), III 2.91 (0.95, 0.40, 0.58, 0.65, 0.33), IV 3.45 (1.13, 0.43, 0.78, 0.78, 0.33). Carapace yellow to green-brown, covered with white scale-like setae on eyes base; fovea longitudinal, short. Chelicerae pale yellow, with two promarginal teeth and one retromarginal tooth smaller than males in size. Legs pale, with three and two pairs of ventral spines on tibiae and metatarsi I, respectively. Abdomen oval, dorsum pale to brown, with irregular brown patches; venter pale.

***Epigyne and vulva*** (Fig. 14A, B): wider than long, with posterior concave; copulatory openings anterior located, almost C-shaped, close to each other; copulatory ducts almost straight, connected to the anterior margins of spherical spermathecae; fertilization ducts originating from the inner-anterior portions of spermathecae.

#### Distribution.

China (Yunnan, Xizang) (Fig. 22A).

### ﻿Genus Stertinius

Taxon classificationAnimaliaAraneaeSalticidae

Simon, 1890

E63B396B-541B-5260-9E88-05EF769A9790

#### Type species.

*Stertiniusdentichelis* Simon, 1890.

#### Comments.

*Stertinius*, is represented by 15 nominal species mainly distributed from East and Southeast Asia (WSC 2024). The genus is poorly defined because the generotype lacks diagnostic drawings, and most of its species were assigned because they present similar habitus and copulatory organs with other known congeners (Prószyński and Deeleman-Reinhold 2013). Based on the above, we assigned the new species to the genus because it generally harbors similar habitus and copulatory organs to *S.ryukyuensis* Suguro, 2020.

### ﻿ Stertinius liqingae sp. nov.

Taxon classificationAnimaliaAraneaeSalticidae

E3C3C2A5-8AAF-54DB-8C64-F4EEC6FABA5F

https://zoobank.org/F6859C70-B45F-4F4B-9875-4369CB957F2D

Figs 15 , 16 , 22B

#### Type material.

***Holotype*** ♂ (TRU-XZ-JS-0071), China: Xizang: Zayu County, Cibagou National Nature Reserve (28°41.43′N, 97°2.86′E, ca 2570 m), 23 Jun. 2023, C. Wang leg. ***Paratypes*** 7♂8♀ (TRU-XZ-JS-0072–0086), same data as for holotype; 5♂3♀ (TRU-XZ-JS-0087–0094), Bowo County, 318 National Highway, nearby the 102 tunnel (30°4.41′N, 95°7.99′E, ca 2160 m), 30 Jun. 2023, C. Wang leg.

#### Etymology.

The specific name is a patronym of Miss Liqing Fan, who helped us collect specimens in Cibagou National Nature Reserve; noun (name) in genitive case.

#### Diagnosis.

**Figure 15.**
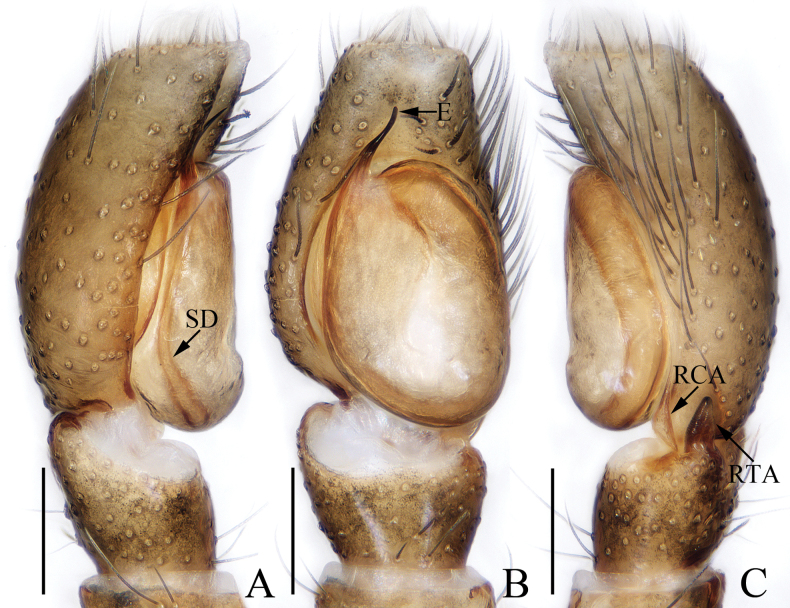
Male palp of *Stertiniusliqingae* sp. nov., holotype **A** prolateral **B** ventral **C** retrolateral. Scale bars: 0.1 mm.

**Figure 16.**
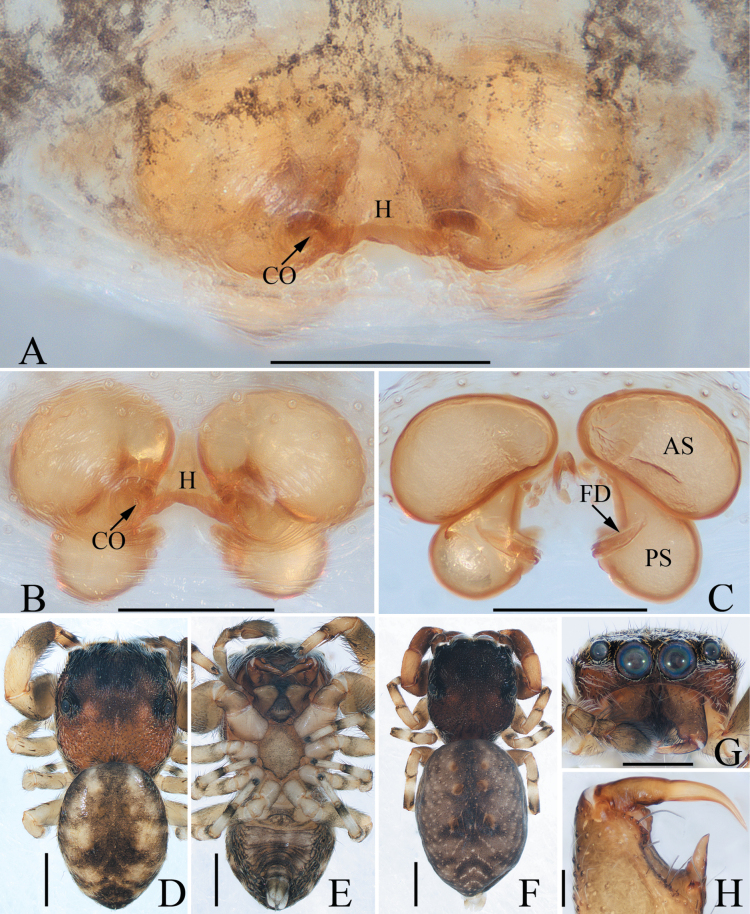
*Stertiniusliqingae* sp. nov., male holotype and female paratype **A, B** epigyne, ventral **C** vulva, dorsal **D** holotype habitus, dorsal **E** ditto, ventral **F** female paratype habitus, dorsal **G** holotype carapace, frontal **H** holotype chelicera, posterior. Scale bars: 0.1 mm (**A–C, H**); 0.5 mm (**D–G**).

*Stertiniusliqingae* sp. nov. resembles that of *S.ryukyuensis* Suguro, 2020 in having similar habitus, papal, and vulva structure, but can be distinguished by: 1) embolus about two times greater than the largest diameter of sperm duct (Fig. 15B), versus more than three times greater than the largest diameter of sperm duct in *S.ryukyuensis* (Suguro 2020: fig. 9); 2) RTA blunt apically in retrolateral view (Fig. 15C), versus pointed in *S.ryukyuensis* (Suguro 2020: fig. 10); 3) epigyne with a triangular hood (Fig. 16A, B), versus absent in *S.ryukyuensis* (Suguro 2020: fig. 12). The male also somewhat resembles that of *Simaethapengi* Wang & Li, 2020 in having similar palpal structure, but is easily distinguished as follows: 1) embolus not curved distally (Fig. 15B), versus curved towards prolaterally in *S.pengi* (Wang and Li 2020: fig. 13C); 2) RTA almost triangular in retrolateral view (Fig. 15C), versus almost bar-shaped in *S.pengi* (Wang and Li 2020: fig. 13B).

#### Description.

**Male** (Figs 15, 16D, E, G, H). Total length 2.59. Carapace 1.38 long, 1.25 wide. Abdomen 1.51 long, 1.13 wide. Eye sizes and inter-distances: AME 0.28, ALE 0.15, PLE 0.14, AERW 1.03, PERW 1.16, EFL 0.70. ***Legs***: I 2.72 (0.88, 0.58, 0.58, 0.40, 0.28), II 1.94 (0.60, 0.38, 0.38, 0.30, 0.28), III 1.74 (0.53, 0.28, 0.35, 0.30, 0.28), IV 2.12 (0.68, 0.33, 0.43, 0.38, 0.30). Carapace almost oval, jacinth to dark brown, setose, with big, irregular dark and brown patches on cephalon; fovea indistinct. Chelicerae yellow to brown, each with two promarginal teeth and one retromarginal pillar-shaped tooth. Endites longer than wide, with straight distal margins. Labium coloured same as endites, bearing several dark setae at anterior margin. Sternum slightly longer than wide. Legs setose, pale to brown, legs I strongest, with enlarged femora, and two pairs of ventral spines on tibia and metatarsi, respectively. Abdomen oval, dorsum brown to dark brown, with irregular pale and dark brown patches, and three pairs of muscle depressions medially; venter pale to brown.

***Palp*** (Fig. 15A–C): tibia wider than long, with straight, tapered, upward extending RTA blunt apically; cymbium ca 1.5 times longer than wide, with lamellar base-retrolateral apophysis; bulb oval, flat; embolus short, originating from ca 10:30 o’clock position of bulb, slightly curved medio-distally and with blunt tip.

**Female** (Fig. 16A–C, F). Total length 2.84. Carapace 1.16 long, 1.01 wide. Abdomen 1.79 long, 1.27 wide. Eye sizes and inter-distances: AME 0.27, ALE 0.14, PLE 0.13, AERW 0.85, PERW 1.00, EFL 0.58. ***Legs***: I 1.96 (0.68, 0.40, 0.40, 0.28, 0.20), II 1.60 (0.50, 0.35, 0.30, 0.25, 0.20), III 1.53 (0.50, 0.28, 0.30, 0.25, 0.20), IV 1.99 (0.63, 0.35, 0.43, 0.33, 0.25). ***Habitus*** (Fig. 16F) similar to that of male except the smaller retromarginal cheliceral tooth and without indistinct pale and pale patches on dorsum of abdomen.

***Epigyne and vulva*** (Fig. 16A–C): wider than long, with central hood between copulatory openings; copulatory openings oval, bilateral to epigynal hood; copulatory ducts short; spermathecae divided into the anterior elliptical chamber and posterior sub-spherical chamber; fertilization ducts originating from the median of the inner portion of posterior chambers, antero-transversely extending.

#### Distribution.

Known only from the type locality in Xizang, China (Fig. 22B).

### ﻿Genus Synagelides

Taxon classificationAnimaliaAraneaeSalticidae

Strand, 1906

B6BB4C06-1DDC-5D6B-9B0C-D18A34AEAAF0

#### Type species.

*Synagelidesagoriformis* Strand, 1906.

#### Comments.

*Synagelides*, contains 72 ant-like species distributed in East, South, and Southeast Asia (WSC 2024). The genus is closely similar to *Pseudosynagelides* Żabka, 1991 in haibuts and copulatory organs, but it can be distinguished from it by the following: 1) the presence of triangular femoral apophysis of palp (for illustration, see Metzner 2024), versus absent in *Pseudosynagelides* (Żabka 1991: figs 9D, 16D); 2) the tegular median apophysis is retrolateral to embolus (for illustration, see Metzner 2024), but prolateral to embolus in *Pseudosynagelides* (Żabka 1991: figs 9B, C, 12A, C, E). It is worth mentioning that *Synagelides* could be much more diverse than its currently known (Wang et al. 2023), and the genus still needs much taxonomic attention, especially the cavaleriei group (see Bohdanowicz 1987), which shares very similar copulatory organs that made it very hard to identify.

### ﻿ Synagelides medog sp. nov.

Taxon classificationAnimaliaAraneaeSalticidae

B82267F2-0C2E-564B-95DC-85DC856E844A

https://zoobank.org/8562AA17-4961-4A70-A222-E39906DF21F7

Figs 17 , 22A

#### Type material.

***Holotype*** ♀ (TRU-XZ-JS-0095), China: Xizang: Medog County, Renqingbengsi scenic area (29°18.10′N, 95°21.29′E, ca 2040 m), 18 Aug. 2023, C. Wang and H. Yao leg. ***Paratypes*** 2♀ (TRU-XZ-JS-0096–0097), same data as for holotype.

#### Etymology.

The species name is a noun derived from the type locality: Medog County.

#### Diagnosis.

**Figure 17.**
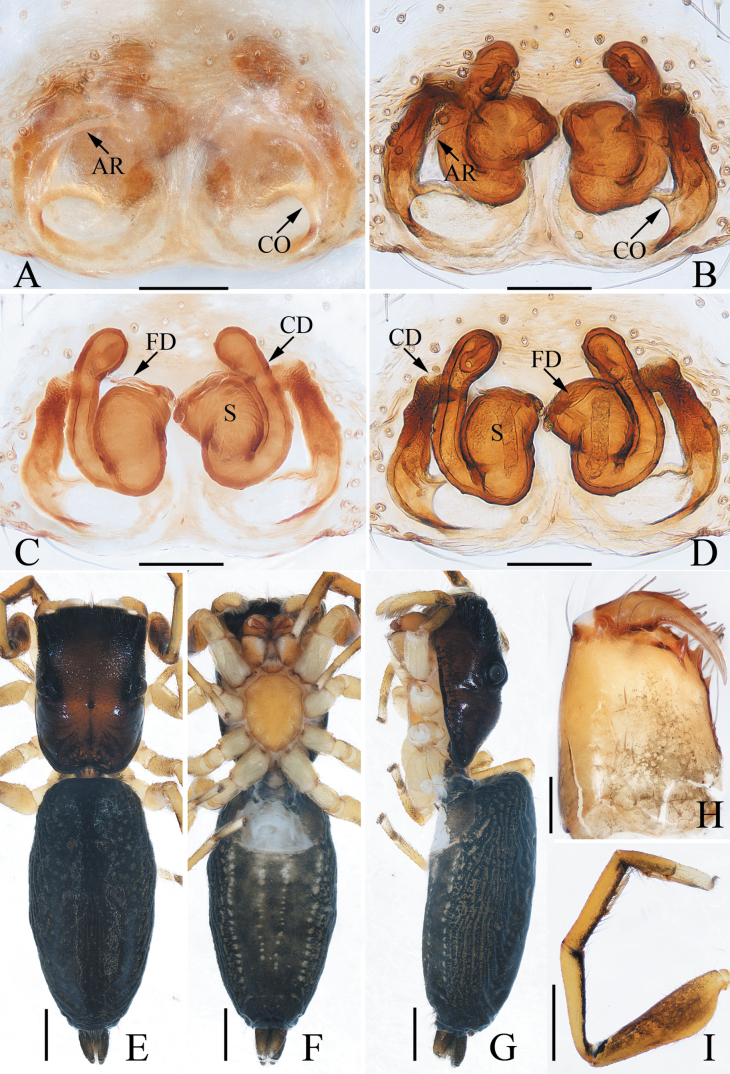
*Synagelidesmedog* sp. nov., holotype **A, B** epigyne, ventral **C, D** vulva dorsal **E** habitus dorsal **F** ditto, ventral **G** ditto, lateral **H** chelicera, posterior **I** leg I, prolateral. Scale bars: 0.1 mm (**A–D, H**); 0.5 mm (**E–G, I**).

*Synagelidesmedog* sp. nov. resembles that of *S.furcatoides* Li, Cheng, Wang, Yang & Peng, 2023 and *S.montiformis* Li, Cheng, Wang, Yang & Peng, 2023 in having similar epigyne and vulva, but can be easily distinguished by the absence of epigynal hood, and mediolaterally located atrial ridge (Fig. 17A–D), versus the presence of epigynal hood, and with the posteriorly located atrial ridge in *S.furcatoides* and *S.montiformis* (Li et al. 2023: figs 4, 5, 13, 26, 34).

#### Description.

**Female** (Fig. 17A–I). Total length 4.06. Carapace 1.56 long, 1.10 wide. Abdomen 2.48 long, 1.24 wide. Eye sizes and inter-distances: AME 0.36, ALE 0.20, PLE 0.19, AERW 1.04, PERW 1.08, EFL 0.84. ***Legs***: I 3.49 (1.13, 0.88, 0.75, 0.45, 0.28), II 2.55 (0.80, 0.40, 0.60, 0.50, 0.25), III 2.78 (0.83, 0.40, 0.65, 0.65, 0.25), IV 3.97 (1.13, 0.53, 1.00, 0.98, 0.33). Carapace almost square, red-brown to dark brown, spotted on eyes field and covered with thin setae anteriorly; fovea hollow. Chelicerae yellow, each with two promarginal teeth and one retromarginal tooth. Endites almost square, with pale inner portions bearing dense brown setae. Labium coloured same as endites. Sternum almost shield-shaped, longer than wide. Legs yellow to brown, with two pairs of ventral spines on tibiae and metatarsi I, respectively. Abdomen elongated, dorsum dark, without indistinct markings; venter paler than dorsum, with pair of dotted lines centrally.

***Epigyne and vulva*** (Fig. 17A–D): wider than long; atrium big, oval, posteriorly located, with pair of arc-shaped, anterolateral ridges; copulatory openings oval, posterolaterally located on atrium, separated from each other by more than their width; copulatory ducts long, slightly curved into C-shaped at anterior half and then posterior extending to connect with the postero-lateral portions of oval spermathecae, with short accessory glands located at the anterior portions of the posterior half; fertilization ducts originating from the antero-inner portion of spermathecae, antero-transversely extending.

**Male**. Unknown.

#### Distribution.

Known only from the type locality in Xizang, China (Fig. 22B).

### ﻿ Synagelides tianquan sp. nov.

Taxon classificationAnimaliaAraneaeSalticidae

6D79F777-FBFF-52F4-B26D-C18EA6913D66

https://zoobank.org/EF1AA08D-17B0-4172-B4FF-F872EB7902C4

Figs 18 , 19 , 22A

#### Type material.

***Holotype*** ♂ (IZCAS-Ar44781), China: Sichuan: Tianquan County (30°2.89′N, 102°45.93′E, ca 730), 7 Jul. 2004, S. Li leg. ***Paratype*** 1♀ (IZCAS-Ar44782), same data as for holotype.

#### Etymology.

The species name is a noun in apposition derived from the type locality.

#### Diagnosis.

**Figure 18.**
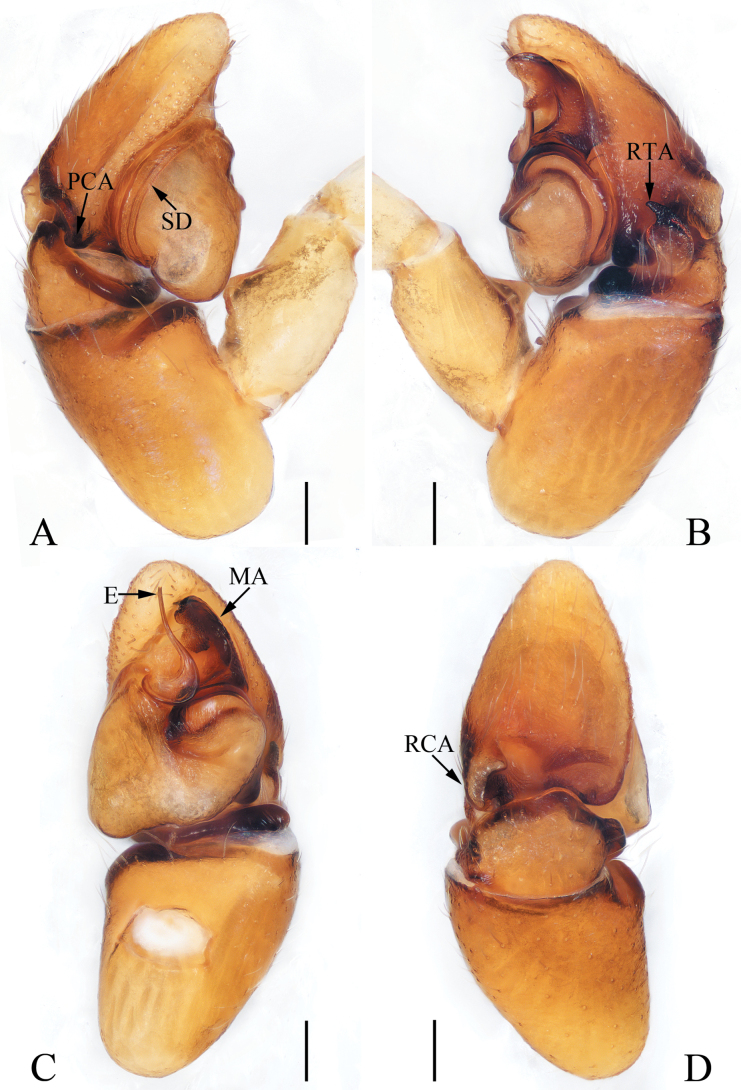
Male palp of *Synagelidestianquan* sp. nov., holotype **A** prolateral **B** retrolateral **C** ventral **D** dorsal. Scale bars: 0.1 mm.

**Figure 19.**
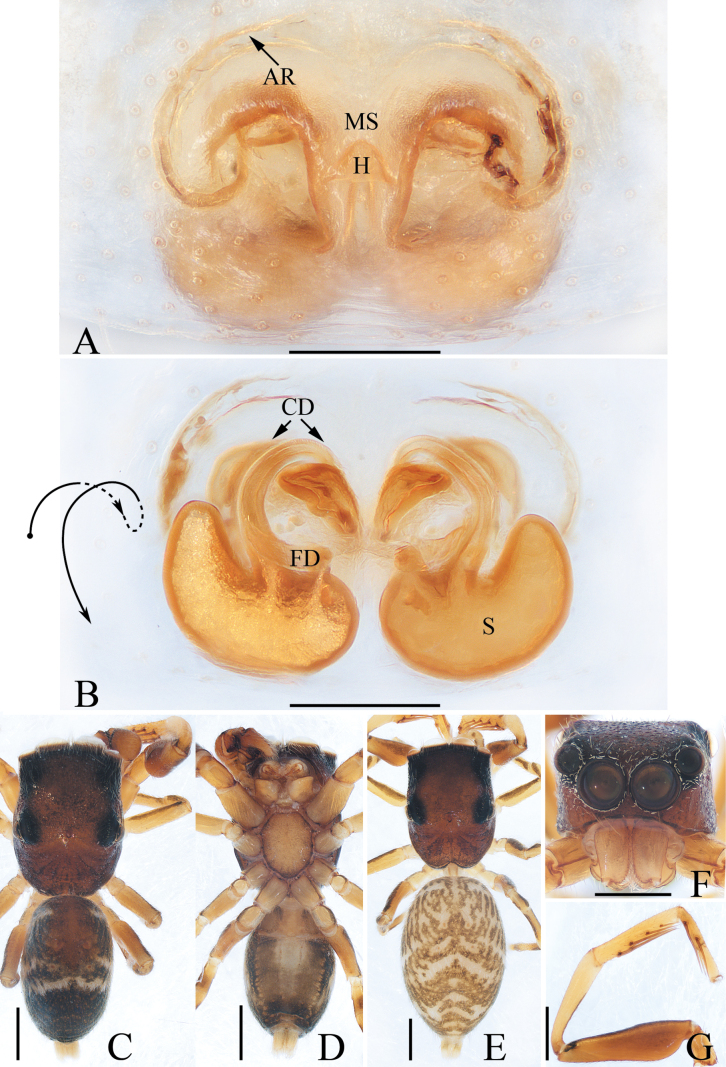
*Synagelidestianquan* sp. nov., male holotype and female paratype **A** epigyne, ventral **B** vulva, dorsal **C** holotype habitus, dorsal **D** ditto, ventral **E** female paratype habitus, dorsal **F** holotype carapace, frontal **G** leg I of holotype, prolateral. Scale bars: 0.1 mm (**A, B**); 0.5 mm (**C–G**).

*Synagelidestianquan* sp. nov. resembles that of *S.emangou* Liu, 2022 and *S.zhaoi* Peng, Li & Chen, 2003 in the general shape of copulatory organs, especially the inverted cup-shaped epigynal hood, but can be readily distinguished as follows: 1) cymbium lacking a flap apophysis (Fig. 18D), versus presence of a big flap apophysis dorsally in *S.emangou* and *S.zhaoi* (Liu et al. 2022: fig. 1C, E–G; Peng 2020: fig. 336c–e); 2) spermathecae curved toward anterior side bilaterally (Fig. 19B), versus not curved in *S.emangou* and *S.zhaoi* (Liu et al. 2022: fig. 2D; Peng 2020: fig. 336g); 3) copulatory ducts strongly curved anteromedially (Fig. 19B), versus almost straight in *S.emangou* and *S.zhaoi* (Liu et al. 2022: fig. 2D; Peng 2020: fig. 336g).

#### Description.

**Male** (Figs 18, 19C, D, F, G). Total length 2.80. Carapace 1.41 long, 1.05 wide. Abdomen 1.41 long, 0.89 wide. Eye sizes and inter-distances: AME 0.33, ALE 0.20, PLE 0.19, AERW 0.99, PERW 1.07, EFL 0.88. ***Legs***: I 3.97 (1.28, 1.03, 0.93, 0.43, 0.30), II 2.36 (0.70, 0.38, 0.55, 0.48, 0.25), III 2.38 (0.70, 0.35, 0.50, 0.58, 0.25), IV 3.33 (1.00, 0.43, 0.80, 0.80, 0.30). Carapace sub-square, red-brown to dark brown, with elevated cephalon and sloped thorax, covered with thin setae; fovea oval, hollowed. Chelicerae pale yellow, each with two promarginal teeth and one retromarginal tooth. Endites almost square, bearing dense pale setae at distal-inner portions. Labium coloured same as endites. Sternum almost shield-shaped, less than 1.5 times longer than wide. Legs yellow to red-yellow, with four and two pairs of ventral spines on tibiae and metatarsi I, respectively. Abdomen elongate-oval, dorsum brown, with two pairs of median muscle depressions, and five transverse pale setal stripes anteriorly and medially, covered by anterior jacinth scutum; venter brown, with pale central area.

***Palp*** (Fig. 18A–D): femur longer than wide, with medio-prolateral, triangular apophysis; patella enlarged; tibia short; RTA weakly sclerotized and broadened base-medially, followed by the acutely narrowed, strongly sclerotized remainder with rather blunt tip directed towards anteroventral side; cymbium longer than wide, with irregular dorsal apophysis and strongly sclerotized prolateral apophysis blunt at terminus; bulb swollen; embolus flat, forming half disc at base and with blunt tip; median apophysis large, irregular.

**Female** (Fig. 19A, B, E). Total length 3.47. Carapace 1.43 long, 1.04 wide. Abdomen 1.96 long, 1.27 wide. Eye sizes and inter-distances: AME 0.35, ALE 0.20, PLE 0.19, AERW 1.00, PERW 1.10, EFL 0.86. ***Legs***: I 3.29 (1.03, 0.78, 0.85, 0.38, 0.25), II 2.31 (0.70, 0.38, 0.53, 0.45, 0.25), III 2.39 (0.73, 0.38, 0.45, 0.58, 0.25), IV 3.44 (1.00, 0.48, 0.88, 0.80, 0.28). ***Habitus*** (Fig. 19E) similar to that of male except paler in color and without setal stripes and scutum on the dorsum of abdomen.

***Epigyne and vulva*** (Fig. 19A, B): wider than long; atrium oval, anterior located, with pair of lateral ridges, and separated by the big, irregular median septum bearing triangular epigynal hood opening downward; copulatory openings beneath the anterolateral portions of median septum; copulatory ducts slender, strongly curved at the position of anterior 1/3; spermathecae boat-shaped, close to each other; fertilization ducts originating from the inner-anterior margins of spermathecae, extending transversely.

#### Distribution.

Known only from the type locality in Sichuan, China (Fig. 22B).

### ﻿Genus Yaginumaella

Taxon classificationAnimaliaAraneaeSalticidae

Prószyński, 1979

8D2EC72E-34FE-5BA6-9FFE-B21FF3C61F43

#### Type species.

*Pellenesususudi* Yaginuma, 1972.

#### Comments.

*Yaginumaella*, one of the members of the subtribe Plexippina Simon, 1901 (Maddison 2015), contains 14 species mainly distributed in East Asia (WSC 2024). The genus has always been considered to be closely related to *Ptocasius* Simon, 1885 (Li et al. 2018; Patoleta et al. 2020), and has even been considered as a synonym of the latter unofficially (e.g. Żabka 1985). One of the influential studies of the two genera is Patoleta et al. (2020), who transferred 37 species of *Yaginumaella* into *Ptocasius* based on the similarities in copulatory organs structures. However, this work has not discussed the difference in habitus patterns (see Li et al. 2018). And now, the generic position of species for these two genera is controversial. Herein, we adopt the view of Li et al. (2018) and assign the new species to *Yaginumaella*.

*Yaginumaellaarmata* (Jastrzębski, 2011), comb. nov. is transferred because it shares a similar habitus and palpal structure to *Yaginumaella* rather than *Pancorius*, given that the embolus originates at the bulb’s base but antero-apically in *Pancorius*. Moreover, the described female of *Y.armata* (new materials collected from Gyirong County, Xizang, were examined by us) is likely mismatched and may belong to a member of the tribe Chrysillini.

### ﻿ Yaginumaella erlang sp. nov.

Taxon classificationAnimaliaAraneaeSalticidae

0AD5605F-6858-5E99-9499-FDC80CBB08E9

https://zoobank.org/AF2DDE2B-5598-41FC-BC67-7BAB8E458136

Figs 20 , 21 , 22B

#### Type material.

***Holotype*** ♂ (IZCAS-Ar44783), China: Sichuan: Tianquan County, Erlangshan National Nature Reserve (30°10.17′N, 102°26.94′E, ca 760 m), 10 Dec. 2004, Z.T. Zhang leg. ***Paratypes*** 2♂2♀ (IZCAS-Ar44784-44787), same data as for holotype.

#### Etymology.

The species name is a noun derived from the type locality: Erlang Mountain National Nature Reserve.

#### Diagnosis.

**Figure 20.**
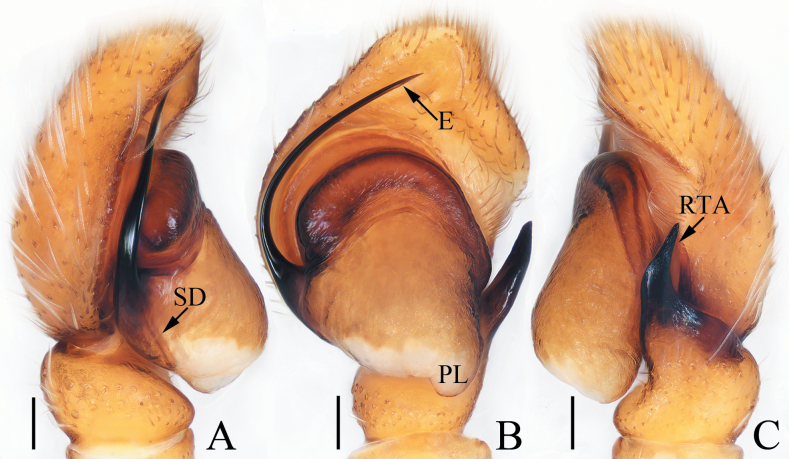
Male palp of *Yaginumaellaerlang* sp. nov., holotype **A** prolateral **B** ventral **C** retrolateral. Scale bars: 0.1 mm.

**Figure 21.**
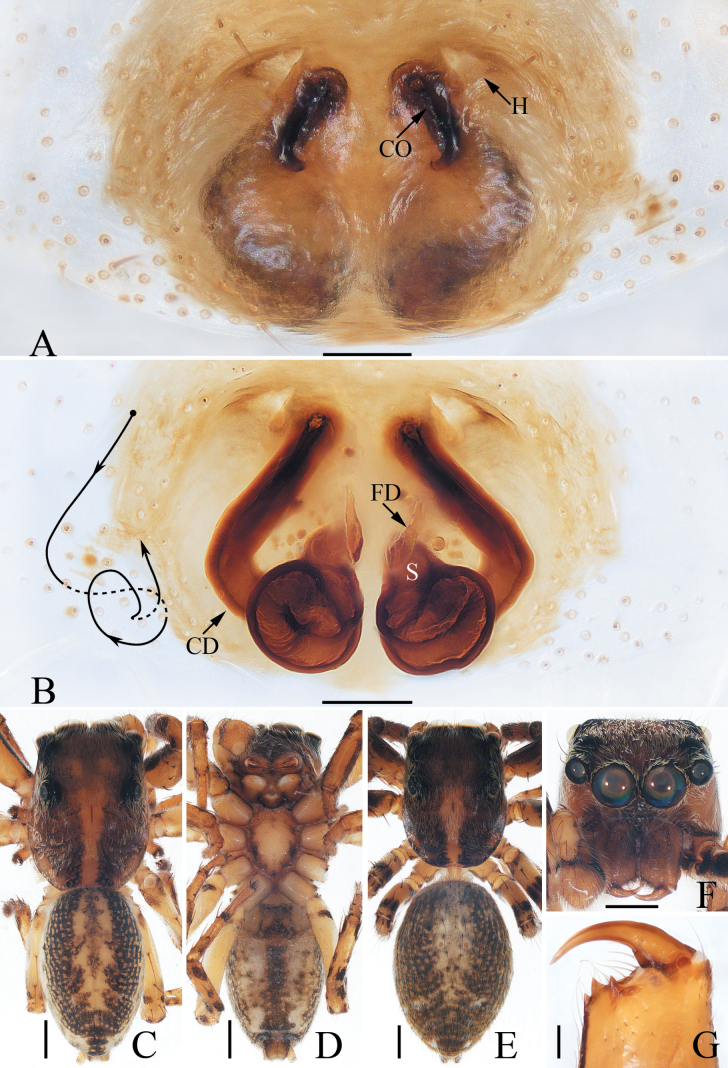
*Yaginumaellaerlang* sp. nov., male holotype and female paratype **A** epigyne, ventral **B** vulva, dorsal **C** holotype habitus, dorsal **D** ditto, ventral **E** female paratype habitus, dorsal **F** holotype carapace, frontal **G** holotype chelicera, posterior. Scale bars: 0.1 mm (**A, B, G**); 0.5 mm (**C–F**).

*Yaginumaellaerlang* sp. nov. resembles that of *Ptocasiuspseudoflexus* (Liu, Yang & Peng, 2016) in general shape of palp, but can be distinguished as follows: 1) RTA curved medially, and with a pointed tip in retrolateral view (Fig. 20C), versus curved distally, and with a rather blunt tip in *P.pseudoflexus* (Liu et al. 2016: figs 15, 16); 2) copulatory ducts extending straight at base in dorsal view (Fig. 21B), versus curved in *P.pseudoflexus* (Liu et al. 2016: figs 15, 16).

#### Description.

**Male** (Figs 20, 21C, D, F, G). Total length 4.27. Carapace 2.11 long, 1.60 wide. Abdomen 2.22 long, 1.36 wide. Eye sizes and inter-distances: AME 0.44, ALE 0.25, PLE 0.22, AERW 1.40, PERW 1.38, EFL 0.91. ***Legs***: I 4.30 (1.30, 0.75, 1.05, 0.70, 0.50), II 3.95 (1.25, 0.75, 0.85, 0.60, 0.50), III 4.25 (1.35, 0.65, 0.90, 0.85, 0.50), IV 4.55 (1.45, 0.65, 0.90, 1.05, 0.50). Carapace red-brown, setose, with longitudinal, central orange-yellow band extending from the middle of eyes field to the posterior margin, and pair of orange-yellow lateral bands; fovea dark red, longitudinal. Chelicerae yellow to brown, each with two promarginal teeth and one retromarginal tooth. Endites paler than chelicerae, slightly widened distally. Labium linguiform, with paler anterior portion. Sternum about 1.5 times longer than wide, tapered at posterior half. Legs pale to dark yellow, with dark stripes prolaterally on femora I, and three and two pairs of ventral spines on tibia and metatarsi I, respectively. Abdomen elongate-oval, dorsum yellow to dark brown, dotted bilaterally, with longitudinal yellow band about one-third the abdominal width; venter pale to dark brown, with longitudinal, central, dark brown band.

***Palp*** (Fig. 20A–C): tibia wider than long, with strongly sclerotized RTA broadened at base, slightly curved medially, and pointed apically; cymbium setose; bulb swollen medio-posteriorly, with small posterior lobe extending postero-prolaterally and blunt at terminus; embolus strongly sclerotized, originating at ca 9 o’clock position of bulb, curved into C-shape base-medially, and with pointed tip directed towards about 2 o’clock position.

**Female** (Fig. 21A, B, E). Total length 4.99. Carapace 2.08 long, 1.60 wide. Abdomen 2.70 long, 1.82 wide. Eye sizes and inter-distances: AME 0.45, ALE 0.27, PLE 0.23, AERW 1.45, PERW 1.43, EFL 0.99. ***Legs***: I 3.80 (1.15, 0.70, 0.85, 0.65, 0.45), II 3.55 (1.15, 0.65, 0.75, 0.55, 0.45), III 4.15 (1.25, 0.65, 0.85, 0.90, 0.50), IV 4.55 (1.40, 0.70, 0.95, 1.00, 0.50). ***Habitus*** (Fig. 21E) similar to that of male except slightly darker in color.

***Epigyne and vulva*** (Fig. 21A, B): slightly wider than long, with pair of anterior sub-triangular hoods open postero-laterally, and anterolateral to copulatory openings; copulatory openings slit-shaped; copulatory ducts long, curved, and twisted; spermathecae indistinct; fertilization ducts lamellar.

#### Distribution.

**Figure 22.**
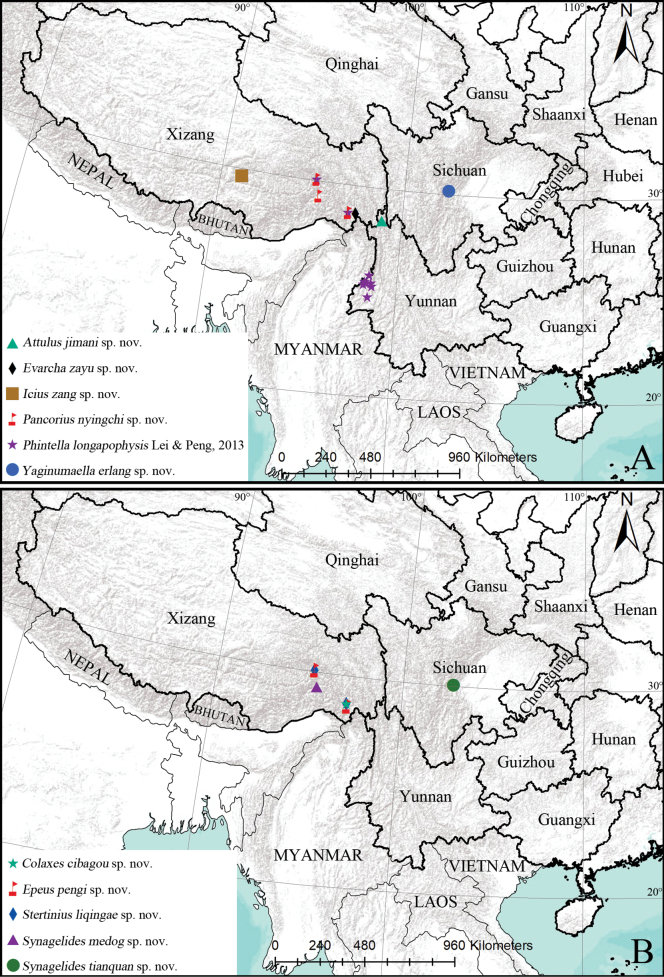
Distributional records of the described species.

Known only from the type locality in Sichuan, China (Fig. 22A).

## Supplementary Material

XML Treatment for Attulus

XML Treatment for Attulus jimani

XML Treatment for Colaxes

XML Treatment for Colaxes cibagou

XML Treatment for Epeus

XML Treatment for Epeus pengi

XML Treatment for Evarcha

XML Treatment for Evarcha zayu

XML Treatment for Icius

XML Treatment for Icius zang

XML Treatment for Pancorius

XML Treatment for Pancorius nyingchi

XML Treatment for Phintella

XML Treatment for Phintella longapophysis

XML Treatment for Stertinius

XML Treatment for Stertinius liqingae

XML Treatment for Synagelides

XML Treatment for Synagelides medog

XML Treatment for Synagelides tianquan

XML Treatment for Yaginumaella

XML Treatment for Yaginumaella erlang

## ﻿Additional information

### Author ORCIDs

Cheng Wang http://orcid.org/0000-0003-1831-0579

Xiaoqi Mi https://orcid.org/0000-0003-1744-3855

Shuqiang Li https://orcid.org/0000-0002-3290-5416

### Data availability

All of the data that support the findings of this study are available in the main text.
